# Biochemical Characterization of a Mycobacteriophage Derived DnaB Ortholog Reveals New Insight into the Evolutionary Origin of DnaB Helicases

**DOI:** 10.1371/journal.pone.0134762

**Published:** 2015-08-03

**Authors:** Priyanka Bhowmik, Sujoy K. Das Gupta

**Affiliations:** Department of Microbiology, Bose Institute, P1/12 C.I.T. Scheme VIIM, Kolkata 700054, West Bengal, India; Molecular Biology Institute of Barcelona, CSIC, SPAIN

## Abstract

The bacterial replicative helicases known as DnaB are considered to be members of the RecA superfamily. All members of this superfamily, including DnaB, have a conserved C- terminal domain, known as the RecA core. We unearthed a series of mycobacteriophage encoded proteins in which the RecA core domain alone was present. These proteins were phylogenetically related to each other and formed a distinct clade within the RecA superfamily. A mycobacteriophage encoded protein, Wildcat Gp80 that roots deep in the DnaB family, was found to possess a core domain having significant sequence homology (Expect value < 10^-5^) with members of this novel cluster. This indicated that Wildcat Gp80, and by extrapolation, other members of the DnaB helicase family, may have evolved from a single domain RecA core polypeptide belonging to this novel group. Biochemical investigations confirmed that Wildcat Gp80 was a helicase. Surprisingly, our investigations also revealed that a thioredoxin tagged truncated version of the protein in which the N-terminal sequences were removed was fully capable of supporting helicase activity, although its ATP dependence properties were different. DnaB helicase activity is thus, primarily a function of the RecA core although additional N-terminal sequences may be necessary for fine tuning its activity and stability. Based on sequence comparison and biochemical studies we propose that DnaB helicases may have evolved from single domain RecA core proteins having helicase activities of their own, through the incorporation of additional N-terminal sequences.

## Introduction

Mycobacteriophages infect and grow in mycobacteria [1]. Several members of the genus *Mycobacterium* such as *M*. *tuberculosis* are pathogenic [2]. A major reason why mycobacteriophages are of interest is because they can be used as tools to study the molecular genetics of mycobacteria [3, 4]. However, in more recent times, there has been a paradigm shift. Mycobacteriophages by themselves are becoming interesting systems to study because of their diversity [5]. With the availability of genome sequences corresponding to a large number of such phages, several comparative genomic studies have been done [6]. These studies have revealed many interesting features about mycobacteriophage genomes and proteomes [5, 7]. It is intriguing that nearly 50% or more proteins encoded by mycobacteriophages have no database matches. Hence, a huge pool of proteins with no known function exists in the mycobacteriophage metaproteome. By characterizing these proteins using either *in silico* or experimental methods or both, it should be possible to discover proteins having novel functions in the near future. The other interesting aspect about mycobacteriophages is genetic mosaicism which is usually caused by illegitimate recombination events [8]. Such events can bring together unrelated DNA segments resulting in the rapid evolution of proteins through, what is sometimes referred to as, domain-lego mechanism [9–11]. Thus, mycobacteriophage derived genes can also serve as useful models to examine how protein functions evolve.

Our research is targeted towards characterizing various mycobacteriophage derived proteins, with the hope of discovering at least a few having novel functions. This endeavour led us to the characterization of a protein (D29Gp65) encoded by gene *65* of mycobacteriophage D29, the product of which turned out to be a novel structure specific nuclease [12]. D29Gp65 and its orthologs belong to a large family of proteins known as the RecA superfamily, named after RecA [13], the major recombinase found in Eubacteria, counterparts of which are also found in the Eukarya (Rad51 and Dmc1) and Archaea (RadA, RadB and aRadC) [14, 15]. All members of the superfamily have a conserved protein fold known as RecA fold which is capable of hydrolyzing nucleotide triphosphates (NTPs) [16]. The energy generated in the process is utilized by the members of this family to execute their respective functions, most of which are linked to DNA transaction processes, such as recombination (RecA) [13], DNA repair (RadA/sms) [17] and unwinding (DnaB) [18]. The domain spanning the RecA fold alone is referred to in this study as the RecA core. Most of the proteins in this family have domains in addition to the RecA core which are apparently necessary to support their specific functions. The notable exceptions are the archaeal proteins RadB and aRadC, and also KaiC, the circadian rhythm regulating protein of cyanobacteria [19], all of which have only the RecA core domain and nothing else [15]. D29Gp65 also belongs to the same category having just the single domain corresponding to the RecA core. The observation that a single domain only protein like D29Gp65 has a specific biochemical function was intriguing. D29Gp65 shares significant homology with another mycobacteriophage encoded protein, Wildcat Gp80 (WCGp80), the subject matter of this study, that was predicted to be a DnaB type helicase. The primary function of helicases is to act as motor proteins to unwind double- stranded DNA, RNA and DNA-RNA hybrids [20]. The energy required for this purpose is derived from the hydrolysis of NTP. The helicases can be classified into six super-families of which DnaB which is the replicative helicase in Eubacteria, belongs to Superfamily 4 (SF4) [20]. DnaB helicases are characteristically hexameric in structure and unwind DNA in an unidirectional manner in the 5'-3' direction [21].

The sequence similarity of the core of WCGp80 with D29Gp65, suggests that it, and by extension all members of the DnaB helicase family, may have evolved from D29Gp65 like proteins. Moreover since a specific biochemical function could be attributed to the single domain protein D29Gp65 therefore it was predicted that the corresponding region of WCGp80 would also have a biochemical function, perhaps similar to that of D29Gp65. To address such possibilities, the present investigation was initiated to biochemically characterize WCGp80 and its core domain. The results presented establish that as predicted, the protein functions as a DnaB class helicase. However, the novel observation presented in this study is that the C-terminal domain (RecA core), when fused to a thioredoxin tag at the N-terminal end supported helicase activity. The observation runs contra to the generally accepted point of view that the core regions of DnaB helicases have no other function apart from acting as NTPases. Based on the phylogenetic and biochemical investigations presented in this study, a model for the evolution of DnaB helicases is proposed.

## MATERIALS AND METHODS

### Bacterial strains, plasmids and phages

*E*.*coli* strains XL1-BLUE, M15 (Qiagen) and Bl21 (DE3) were used for the cloning and expression of recombinant genes respectively. Mycobacteriophage Wildcat was a kind gift from Dr. Graham F. Hatfull, University of Pittsburgh.

### Chemicals

Label-free oligonucleotides as well as 6-FAM and black hole quencher labeled oligonucleotides were purchased from Eurofins Genomics India Pvt. Ltd. NTPs were purchased from Sigma (USA). Radiolabeled nucleotide γ - ^32^P ATP (specific activity 3000 Ci/mmol) was obtained from BRIT, Mumbai, India. Ni^2+^- nitrilotriacetic acid (NTA) agarose, used for the affinity chromatographic preparation of His_6_-tagged proteins, was purchased from Qiagen (Valencia, CA). Restriction enzymes and DNA-modifying enzymes such as polynucleotide kinase were purchased from New England BioLabs (NEB). Sephacryl S-200 size exclusion column material was purchased from Sigma-Aldrich.

### Phylogenetic analysis

Orthologs of WCGp80 were identified by database search using the BLASTP program. CLUSTALW [22] based alignments of multiple sequences were performed using MEGA 5.2 software [23]. Pairwise and multiple-alignments were performed with gap opening and extension penalties of 5 and 0.1 respectively, or as stated. The weight matrices chosen were either PAM or BLOSUM. The alignments created with MEGA 5.2 were subsequently processed using the Bioedit sequence alignment editor or Geneious for convenient schematic representations. Structure-based alignments were reproduced using Espript 3. Using these alignments, neighbour-joining trees [24] were constructed with MEGA5.2 [23]. Bootstrap analysis was performed using 1000 replicates.

### Cloning and expression of Mycobacteriophage Wildcat gene *80* and its mutants

For biochemical characterization of WCGp80, the gene encoding it was PCR amplified, using primers 5' CGGGATCCGTGCCAGCCTACGACGAGC 3' (forward) and 5' CCCAAGCTTTTATGCCACAGCTTCATCATTATGC 3' (reverse) from mycobacteriophage Wildcat genomic DNA and subsequently cloned into the BamHI- HindIII site of the expression vector pQE30 (Qiagen). The underlined nucleotides in the primers indicate restriction enzyme cleavage sites. The recombinant protein synthesized from the plasmid thus constructed had a His_6_ tag at the N-terminal end. To generate the N–terminal truncated version of the protein, the coding region corresponding to the 189^th^ to 441^st^ amino acid residue (C- terminal end) of WCGp80 was PCR amplified using primers 5' CGGGATCCATGCTTGTGGTGGGTGCCC 3' (forward) and 5' CCCAAGCTTTTATGCCACAGCTTCATCATTATGC 3’ (reverse) and cloned in the BamHI-HindIII restriction sites of pET32a vector in frame with a thioredoxin tag provided in the vector. This thioredoxin (Thio) tagged, truncated version of the protein, Thio-ΔN(1–189)WCGp80, which lacks the N-terminal sequence from 1–189, also has a His_6_ tag which was utilised for affinity purification following its synthesis in *E*.*coli* BL21 (DE3) cells. Mutation (K, the 201^st^ amino acid to A) was introduced into the Walker A motif of WCGp80 and its truncated version Thio-ΔN(1–189)WCGp80 by Quick-Change site-directed mutagenesis kit (Stratagene, Canada) according to manufacturer’s instructions using the primers: 5' CCCGGTTGCGGCGCGACGGTTTTCGTT 3' (forward) and 5'AACGAAAACCGTCGCGCCGCAACCGGG 3' (reverse). The mutant proteins derived, were named as either WCGp80(K201A) or Thio-ΔN(1–189)WCGp80(K201A) depending on which polypeptide was targeted, the Wild type or the truncated version. The K201A nomenclature was used in both cases to emphasize that the same K residue in the Walker A motif was mutated.

### Purification of recombinant proteins

The pQE30 and pET32a expression constructs generated as described above were transformed into *E*. *coli* M15 or BL21 (DE3) respectively. Cultures were grown in 2XYT medium supplemented with 100 μg/ml of ampicillin at 37°C. At an O.D._600_ (optical density at 600 nm) of 0.4, isopropyl-β-D-1-thiogalactopyranoside (IPTG) was added to a final concentration of 0.5 mM to induce His-tagged protein synthesis at 28°C for 3 h. Bacterial pellets obtained by centrifugation at 10,000g for 20 min were stored at -80°C until use. His_6_-tagged recombinant proteins were affinity purified using Ni^2+^-nitrilotriacetic acid (NTA) agarose chromatography under native conditions according to standard protocol (Qiagen). Cells harvested by centrifugation were lysed by sonicating in buffer A (50 mM Tris-HCl (pH 8.0),100 mM NaCl, 10 mM imidazole and 10% glycerol). After centrifugation at 30,000 g for 10 minutes the clear supernatant was loaded onto a 1 ml Ni^2+^-NTA agarose column pre- equilibrated with buffer A. The column was washed with 10 column volumes of buffer B (100 mM Tris-HCl [pH 8.0], 500mM NaCl, 20 mM imidazole and 10% glycerol). The bound protein was eluted with buffer C (100 mM Tris-HCl [pH 8.0], 50 mM NaCl, 250 mM imidazole and 10% glycerol) and analysed by 12% sodium dodecyl sulphate polyacrylamide gel electrophoresis (12% SDS-PAGE). Fractions containing more than 90% pure protein were pooled and dialyzed overnight at 4°C against buffer D (40 mM Tris-HCl [pH 7.5], 50 mM KCl, 7 mM β-Me, 1 mM EDTA and 10% glycerol).

### Size Exclusion Chromatography

Affinity-purified proteins were further purified by loading 0.5 ml of the sample onto a size exclusion gravity flow column (Sephacryl S-200 column, Sigma-Aldrich), having a bed volume of 40 ml and eluting with a buffer comprising 40mM Tris–HCl, pH 7.5, 50 mM KCl, 1mM EDTA, 10% v/v glycerol. The void volume of the column was determined using the high molecular weight marker Blue dextran. The column was run at a flow rate of 0.5ml/min. Fractions (0.5 ml) were collected and analysed by 12% SDS-PAGE. Fractions containing purified protein were pooled, aliquoted and stored at -20°C. The protein concentrations were determined by Bradford assays, according to the manufacturers’ instructions (BioRad), using bovine serum albumin (BSA) as a standard.

For accurate determination of molecular weight (Mw), samples containing the protein in a highly purified form were loaded on a Superose 6 10/300 GL, size exclusion column (GE Healthcare) and subjected to separation in a FPLC equipment at a fiow rate of 0.4 ml/min using a buffer containing 40 mM Tris-HCl (pH 7.5), 50 mM KCl, 2 mM dithiothreitol. Standards used in this experiment were, Thyroglobulin (670 kDa), β-Amylase (200 kDa), Bovine serum albumin (66 kDa), Ovalbumin (44 kDa), and Cytochrome C (12.5 kDa). The logarithms of the Mw values of the standards were plotted against the elution fraction number, and the standard curve that emerged was used to accurately assign a specific Mw to the protein in its native form.

### Preparation of DNA substrates for helicase assays

The sequences of the oligonucleotides used as DNA substrates are given in S1 Table. The same oligonucleotides were used in our earlier study involving D29Gp65 [12]. Complementary oligonucleotides were labeled and annealed using standard protocols. Briefly, T4 polynucleotide kinase was used to phosphorylate the 5' end of one of a pair of oligonucleotides required for the formation of the fork (specified in S1 Table) with γ ^32^P-ATP (BRIT, Mumbai, India). The labeling was done in such a way that the γ ^32^P tag was placed at the stem end of the fork. Annealing reactions were carried out by mixing 100 pmoles each of the complementary oligonucleotides, one of which was labeled, in 50μl annealing buffer (40 mM Tris-HCl [pH 7.5], 80 mM NaCl, 10 mM MgCl_2,_1 mM DTT), followed by heating to 95°C for 2.5 minutes and gradual cooling to room temperature. The annealed products were subsequently purified by PAGE using the “crush-and-soak” method as described previously [25]. The oligonucleotides used for fluorescence based helicase assay are also indicated in S1 Table. For fluorescence based helicase assay, 100 pmoles of 6-FAM labeled strand was mixed with 100 pmoles of Black Hole Quencher1 labeled strand in the same annealing buffer as stated above. Oligonucleotides were annealed by the same process as mentioned earlier. The annealed substrate was directly used for helicase assay. As in the case of the radiolabel, the fluorescence tags were also positioned at the stem end of the fork.

### DNA unwinding assay

DNA unwinding was monitored using radio as well as fluorescence labeled fork substrates. ^32^P-labeled fork substrates (S1 Table) (1 nM) and the protein (220 nM or as mentioned) was added to the reaction buffer containing 50 mM Tris-HCl (pH 8.0), 50 mM KCl, 2 mM dithiothreitol, 0.25 mg of bovine serum albumin/ml, 4 mM MgCl_2_, 4 mM ATP, and 4% sucrose (buffer E). After incubation at 37°C for 10 min, the reactions were stopped with 5X stop solution (1.25% SDS,75 mM EDTA, 25% glycerol) and resolved by 12% native PAGE. Boiled substrates were loaded into control lanes to identify the bands corresponding to the un-annealed labeled oligonucleotide. The gels were dried, and bands were visualized by autoradiography. Wherever necessary the band intensities were quantitated using a Versadoc gel documentation system (Biorad laboratories).

The DNA substrate used for fluorescence based helicase assays was generated as described above. Annealing reactions were carried out by mixing 100 pmoles each of the complementary oligonucleotides, one of which was labeled with 6-FAM fluorophore and the other with blackhole quencher-1. Before DNA unwinding, the two molecules are in close proximity and the emission of 6-FAM is low due to FRET between the two molecules. Upon unwinding, the 6-FAM emission is enhanced due to the disruption of FRET as the two DNA strands are separated. The change of 6-FAM emission was monitored for 600 seconds in real time using Hitachi F-7000 spectrofluorometer to observe the unwinding process. 6-FAM was excited at 492 nm (5 nm bandwidth) and emission was monitored at 520 nm (5 nm bandwidth). All the assays were performed at 37°C.

### Fluorescence quenching experiments

The intrinsic tryptophanyl fluorescence of WCGp80 was first determined using a Hitachi F-7000 spectrofluorometer. The excitation and emission wavelengths were 280 and 340 nm respectively. Excitation and emission slits were set to the spectral width of 5 nm. Fluorescence quenching experiments were performed as described in a previous investigation [26]. Titrations were done by adding increasing amounts of the desired oligonucleotide to the protein sample at the concentration mentioned. The ratio between the fluorescence observed in the presence of the oligonucleotide (F) and that in its absence (F_0_) was plotted against oligonucleotide concentration.

### ATPase assay

ATPase activity was measured using a malachite green based colorimetric assay in which inorganic phosphate produced by ATP hydrolysis is monitored [27]. Reactions were carried out in reaction buffer containing 50 mM Tris-HCl (pH 8.0), 50 mM KCl, 2 mM dithiothreitol, 0.25 mg of bovine serum albumin/ml, 4 mM MgCl_2_, and 4% sucrose (buffer E) in the presence of desired amounts of single stranded (ss) DNA (oligonucleotides of defined length), and ATP. Reactions were initiated by adding the enzyme and ATP. The reaction mix was incubated at 37°C for 15 minutes. 10 μl of 0.1M EDTA was added to terminate the reaction. Samples were subjected to malachite green assay along with a series of NaH_2_PO_4_ dilutions ranging from 1 to 10 mM, which was included for the purpose of calibration.

### Dynamic light scattering (DLS)

DLS experiments were performed using a Malvern Zetasizer Nano instrument to estimate the size of the protein. The buffer used for measurements was 50 mM Tris–HCl (pH 8.0), 50 mM KCl, 2 mM DTT and 1mM MgCl_2_. The protein concentration used was 1μM unless otherwise stated. The samples were passed through a 0.22 μm filter prior to analysis. Auto-correlations were incrementally stored every 10s at a temperature of 25°C. The data were analysed with the Zetasizer Nano software for determination of hydrodynamic radius. In the case of polydisperse samples, the weight average radius was considered for interpretation.

## Results

### Phylogenetic analysis identifies WCGp80 to be related to an ancestor of DnaB helicase family proteins

**Fig 1:**
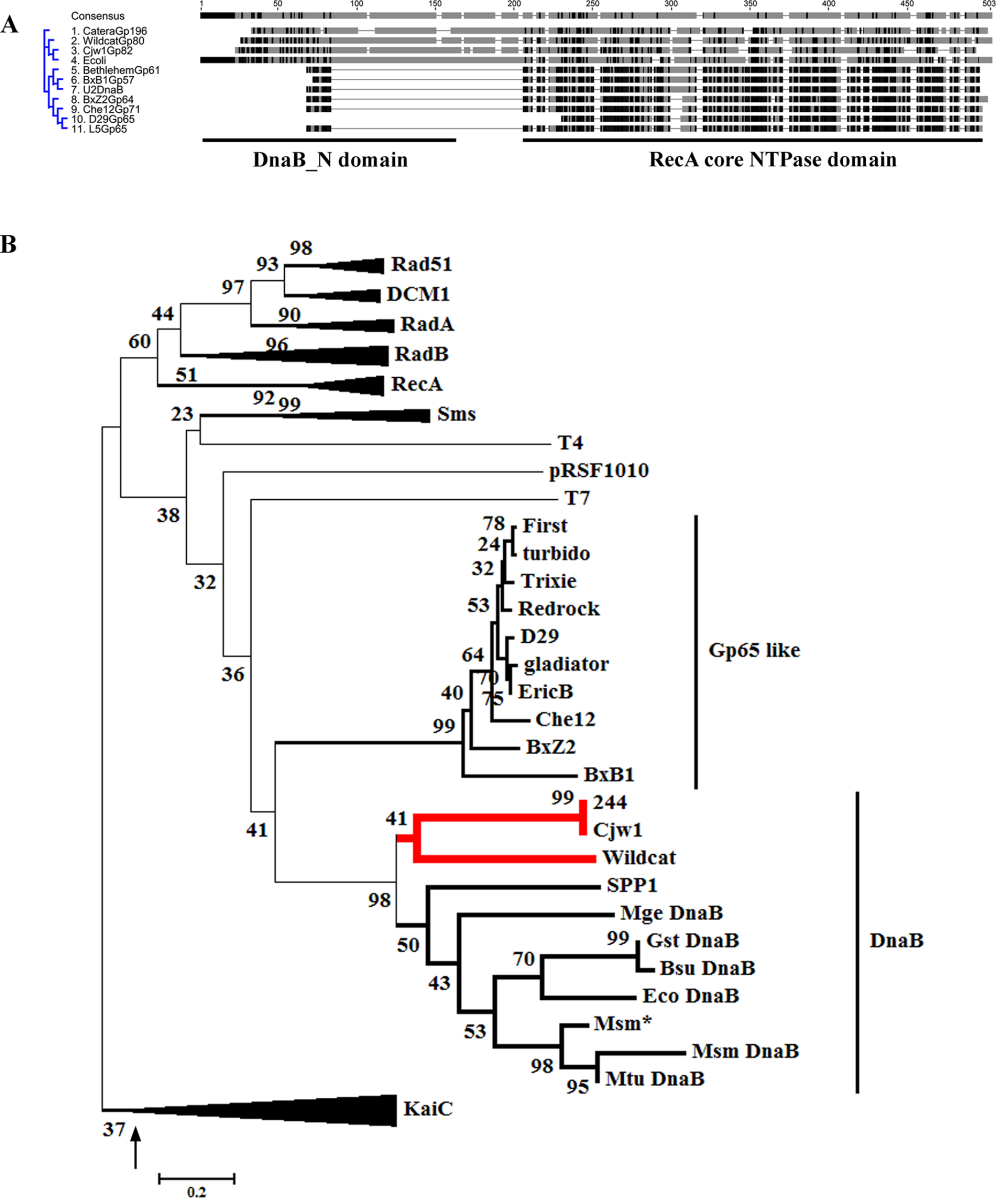
Evolutionary analysis of WCGp80. **(A)** Alignment of Mycobacteriophage encoded DnaB helicase family proteins using Geneious 7.1.4. The *E*. *coli* DnaB was used as the reference. Of the various mycobacteriophage proteins represented in the alignment, those from Catera, Wildcat and CjW1 were full length DnaB helicases, having both the DnaB N and C-terminal (RecA NTPase core) domains (as indicated below) whereas the rest contained principally only the C-terminal RecA NTPase core. Aligned residues are indicated in shades of grey, the most conserved being the darkest. The clustering pattern is shown on the left. **(B)** Phylogenetic tree derived from an alignment of the core domains of various members of the RecA family (refer S1 and S2 Figs for details). The list includes DnaB helicases from Eubacteria and their phages, D29 Gp65 like single domain proteins, Circadian Rhythm regulating proteins (KaiC), eubacterial DNA repair proteins RadA/Sms, and several recombination related proteins of either eubacterial, eukaryal or archaeal origin, RecA,Rad51/DCM1 and RadA respectively. The WCGp80 branch is indicated by thick red lines. The evolutionary history was inferred using the Neighbor-Joining method. The percentage of replicate trees in which the associated taxa clustered together in the bootstrap test (1000 replicates) is shown next to the branches. The tree is drawn to scale, with branch lengths in the same units as those of the evolutionary distances used to infer the phylogenetic tree. The arrow indicates the branch on which a root may be placed to convert the tree into a rooted one. The various clusters are indicated on the right. Expanded versions of the collapsed branches are shown in S2 Fig.

Gp65 of Mycobacteriophage D29 is an independently existing RecA core domain protein, that was functionally characterized earlier to be an exonuclease [12]. To identify its homologs, a BLAST analysis with the D29Gp65 sequence was performed. Several protein sequences, highly homologous to D29Gp65, as judged by the low Expect (E) values (< 0.001), were identified, all of which were of mycobacteriophage origin. The identified homologs were of two types: (i) Gp65 like, having only the RecA core domain and (ii) DnaB like, in which an additional N-terminal domain was present (Fig 1A). DnaB helicases are the replicative helicases of Eubacteria. Replicative helicases of organisms belonging to the two other domains of life–Archaea and Eukarya are evolutionarily unrelated to DnaB (non-orthologous) and do not belong to the RecA superfamily [14, 28].

Most of the DnaB helicases incorporated in the detailed phylogenetic analysis are the ones about which at least some biochemical information is available. In particular, the DnaB helicase of *E*. *coli*, has been extensively studied with respect to its domain structure [18]. Since this investigation is about a bacteriophage helicase, therefore, several well-studied DnaB type helicases from bacteriophages were included. These are G40P from SPP1, a *Bacillus subtilis* phage, Gp41 from the coliphage T4 and Gp4 from T7, also a coliphage [29–31]. The helicases of *M*. *tuberculosis* and *M*. *smegmatis* were incorporated in the tree as WCGp80 is derived from a phage that infects mycobacteria and a possible scenario that the mycobacterial and mycobacteriophage helicases are closely related had to be examined. The *M*. *smegmatis* protein has not been investigated so far by any group, but its *M*. *tuberculosis* counterpart has been characterized [32]. In *M*. *smegmatis* mc^2^155 a gene encoding a single domain RecA core protein could be identified (Accession no. AFP40951.1) that was homologous to the C-terminal domain of the full-length DnaB encoded by the same organism (Accession no. AFP4315135). The putative protein indicated as Msm* in the phylogenetic tree, (Fig 1B) is approximately of the same length as that of Gp65. To understand the phylogenetic affinity of this protein, its sequence was also incorporated. Several other sequences were incorporated, which were selected randomly.

Apart from the sequences of DnaBs and D29Gp65 like proteins, those of many other proteins of the RecA superfamily were included. The list includes RecA itself, the hallmark protein of this family, and the RadB family proteins, which are recombinases found in Archaebacteria [15]. The RadB proteins, although they are from Archaea are highly relevant to the present study as they have single domain structures similar to D29Gp65. It has been proposed that these proteins represent the extant version of an ancient DNA repair protein, and thus, they have been named as ‘mementos from the last universal common ancestor’ [33]. The precise function of RadB is not known although evidences indicate that PK-REC, a RadB family protein from the archaeon *Pyrococcus* sp. KOD1 may function as a nuclease [34], an observation that has some similarity with that made using D29Gp65 reported in our previous investigation [12]. Whatever may be the mechanism it appears that RadB family proteins assist RadA the archaeal RecA homolog in bringing about recombination [35].

The pRSF1010 replication protein, RepA which functionally is a helicase was considered for the analysis [36]. This helicase has a conserved RecA core, like DnaB, but unlike it, its N-terminal domain is shorter, only 30 amino acid residues, as compared to that of DnaB, which is about 200 amino acid residues long. Hence, pRSF1010 RepA may be considered as a reduced version of DnaB helicase [37].

Other non-DnaB helicase members included are (RadA/Sms) which play an important role in bacterial DNA repair [17] and the circadian rhythm protein KaiC [38]. KaiC is not typically a DNA transaction related protein. It is involved in the generation of circadian rhythm in cyanobacteria, in partnership with KaiA and B [38]. That KaiC is involved in a DNA transaction unrelated function was considered to be initially surprising. However, it was demonstrated that KaiC, like many of its counterparts within the RecA family, forms hexamers and binds to DNA forks [19]. The evolutionary origin of KaiC is a debatable issue. Although KaiC is a Eubacterial protein it has a strong sequence similarity with aRadC, a single domain RecA core protein of Archaeal origin which has DNA repair- related properties. It has been proposed that KaiC proteins may have originated from aRadC type ancestors [15]. Apart from the ones that have been described above, a number of other RecA cores belonging to proteins having diverse functions were included. The list includes the eukaryotic homologs DCM1 and RadA. The choice of the members in case of the non-DnaB clades is to some extent, arbitrary although conscious efforts were made to include members that have been used earlier to create a RecA evolutionary tree [14].

A phylogenetic tree created based on the alignment (S1 Fig) revealed that the Gp65 homologs comprised a novel clade (Fig 1B, and S2 Fig). One can place a root on the KaiC branch to create an out-group, as KaiC proteins possibly are of archaeal origin, and therefore, they must have diverged away from the eubacterial RecA family members in the distant past [15]. By creating such an out-group, it is possible to obtain insight into the evolutionary origin of Gp65 like single domain RecA core proteins and DnaB helicases. From the rooted tree thus constructed, it appears that Gp65 and DnaB helicases may have evolved from a common ancestor (Fig 1B). Furthermore, we found that the DnaB helicase proteins derived from mycobacteriophages Catera, CjW1 and Wildcat have emerged from a node that represents a possible ancestor of the DnaB helicase family. It is also evident that these mycobacteriophage derived proteins root deep in the DnaB helicase branch indicating that they possibly represent extant versions of an ancient DnaB helicase (Fig 1B). Except for the bacillus phage SPP1 helicase G40P which fits well in the DnaB cluster, the other phage (T7 and T4) or plasmid (pRSF1010) derived members appear as singletons with no apparent affinity for any of the groups. The general conclusion that emerges from this phylogenetic analysis is that Gp65 like single domain proteins and DnaB helicases are evolutionarily closely linked and may have evolved from a common ancestor.

### WCGp80 is a helicase

**Fig 2:**
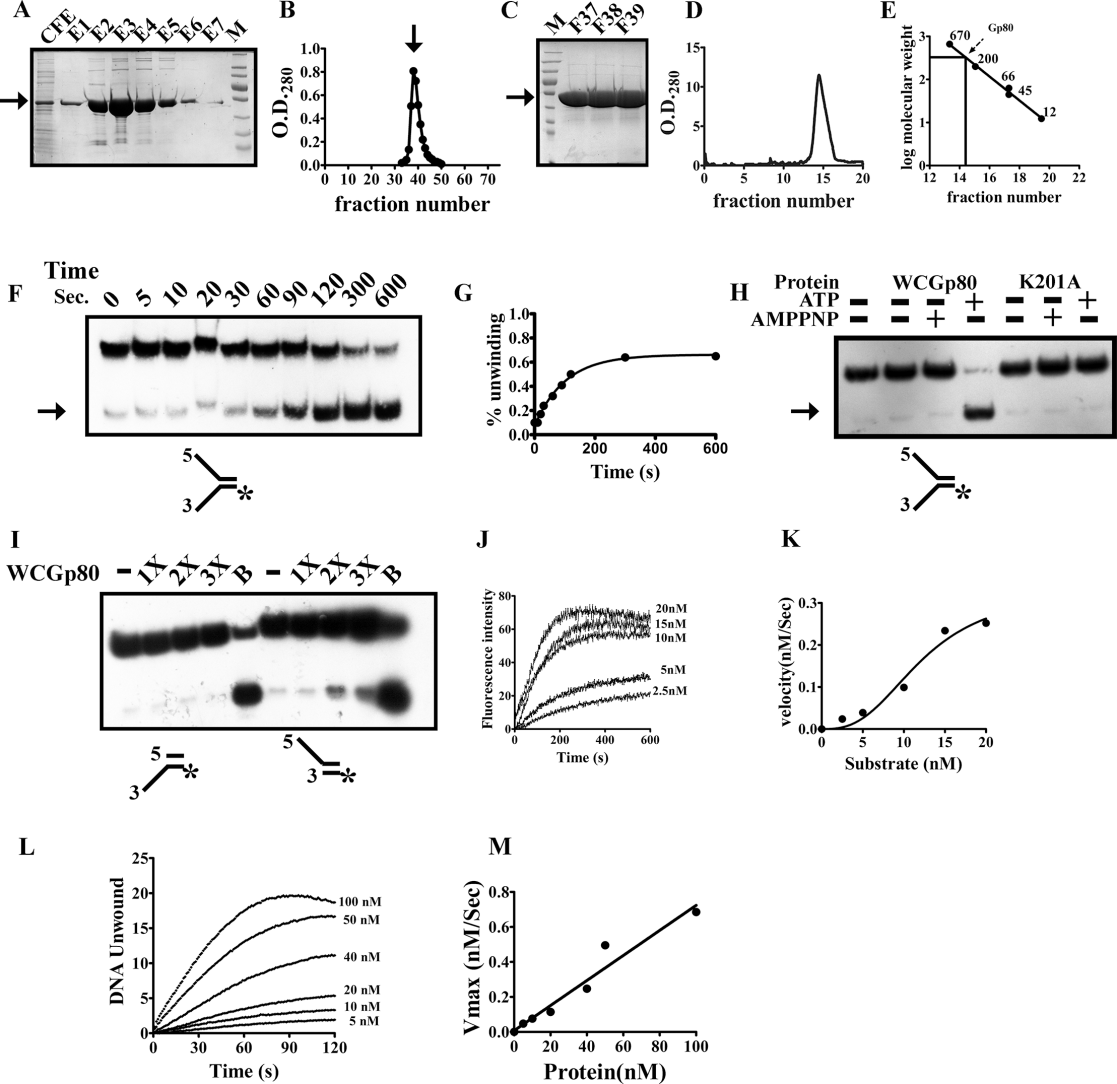
Expression, purification and initial characterisation of WCGp80. **(A)** 12% SDS-PAGE analysis of the affinity purified recombinant WCGp80 protein. The lanes marked CFE and E1-E7 represent cell free extract and the elution fractions respectively. **(B)** Purification of WCGp80 using Size exclusion chromatography. The fractions enriched in WCGp80 after Ni-NTA purification were pooled and passed through the Sephacryl S-200 size exclusion column**.** The peak position corresponding to the void volume marker Blue Dextran (2000 kDa) is indicated by an arrow. **(C)** The fractions corresponding to the single peak were analysed by performing 12% SDS-PAGE to confirm the presence of the desired protein **(D)** These protein fractions were pooled and an aliquot of the sample thus obtained was again loaded onto an analytical column, Superose 6. **(E)**The Mw of WCGp80 (indicated simply as Gp80 only), which eluted as a single peak was estimated on the basis of a standard curve**.** The standards used are mentioned in the Materials and Methods section. **(F)** Autoradiogram of 12% native gels showing time dependent release of ^32^P labeled strand (indicated by an arrow on the left) from the fork substrate. The concentration of the enzyme used was 120 nM and fork substrate, 1 nM. Reaction times are shown above the autoradiogram. **(G)** The % unwinding was plotted versus time. The solid lines represented the fit of the data to the equation Y = Y0 + Ymax*(1-exp (-K*x) (refer S3 Fig for explanation), where **Y** is the conversion at any time point (**x**), **Y0**, the initial value, **Ymax** the final value reached and K, the observed first-order rate constant. **(H)** Autoradiogram of helicase assays performed using the substrate mentioned in **(F)**, in the presence or absence of co-factors mentioned above. The experiment was performed both with WCGp80 and WCGp80(K201A), the latter being indicated as K201A. **(I)** Autoradiogram of helicase assays performed using the incomplete fork structures shown below using increasing concentrations of WCGp80. 1X corresponds to 220 nM of the protein. B, stands for ‘boiled’ fork structures in this experiment and others to follow. Non- radioactive FRET based assays were performed to study the substrate saturation kinetics of WCGp80 **(J-M).** Initial velocities of helicase action derived from the linear portion of the traces shown in **(J)** were plotted against substrate concentrations **(K).** The data points were then fitted to a Hill equation, V = Vmax*X^h/(K' + X^h), where V is the observed initial velocity at a particular fork concentration, **Vmax** is the maximal velocity, **K'** is the Hill constant and **h** the Hill coefficient**. (L)** Time course of helicase reaction using different WCGp80 concentrations as indicated keeping the substrate at saturating level (80 nM). The Vmax at each protein concentration was determined from the slope of the linear portion of the graphs. **(M)** A plot of V_max_ versus protein concentration. The slope of the line connecting the points gives the K_cat_.

WCGp80 is a 52 kDa protein that was predicted by bioinformatic analysis to be a DnaB helicase. DnaB helicases which are the replicative helicases of Eubacteria are known to unwind DNA in a 5'-3' direction at replication forks. To biochemically characterize WCGp80, a hexa—histidine tagged version of the protein was synthesized in *E*. *coli*. The synthesized protein was soluble and could be purified in the native form by affinity chromatography (Fig 2A). The affinity purified fractions were highly enriched in the desired protein, and contaminating bands were either absent or present at a very low level (Fig 2A, elution fractions E1-E6). The eluted fractions were pooled and loaded on to a preparative scale size exclusion chromatography (SEC) gravity flow column packed with Sephacryl S-200 beads. The protein eluted in the void volume (Fig 2B) indicating that it exists as a multimer, the size of which is larger than 200 kDa, the exclusion limit for the column. The highly pure protein sample (Fig 2C) obtained after SEC through Sephacryl S-200 was used in all the experiments meant to characterize the biochemical properties of WCGp80. To obtain a precise estimate about the oligomeric state, an aliquot of the sample was resolved on a Superose 6 column, which has a larger resolving range (5–5000 kDa), as compared to Sephacryl S-200. A single major peak (Fig 2D) was observed following elution. By calibrating the column with standard markers, it was possible to accurately determine the Mw of the protein (Fig 2E). The Mw was deduced to be 318 kDa. Considering a monomeric molecular weight of 52 kDa, we conclude that the protein exists as a hexamer, which is consistent with the fact that the members of the DnaB family of helicases are mostly hexameric [39].

Phylogenetic analysis predicted that WCGp80 is a DnaB helicase family protein. Hence, a helicase assay was designed to test its function. The assay involves the use of a DNA fork substrate labeled at 5΄end (S1 Table). DnaB helicases prefer forks as substrates, and hence such a structure was a natural choice. However, a DNA fork can be a substrate for many other enzymatic reactions, including structure specific nuclease [12], recombinases [40] and even KaiC [19]. Thus, while the fork based assay designed is targeted to unravel the helicase activity of WCGp80, other fork related functions, if any, associated with this protein were expected to surface. Such a strategy worked out well in our previous study with D29Gp65, where a structure specific-nuclease activity was unearthed while performing an assay using the helicase protocol [12].

The ability of WCGp80 to unwind forked duplex was monitored kinetically under single turnover conditions (Fig 2F), in which the enzyme (WCGp80) is present in far excess as compared to the substrate [41]. In this condition, all the substrate molecules may be assumed to be bound to the protein. That such an assumption is correct was verified by determining the affinity of the enzyme (WCGp80) for the fork substrate using a fluorescence polarization assay (S3 Fig). The assay yielded a K_d_ of 9.5 nM. If the concentration of the enzyme used is 120 nM and the substrate 1 nM, then it can be demonstrated that at equilibrium only a negligible fraction of the fork (1% at the most) would remain unbound, the rest of it (99%) will be in the bound state (S3 Fig explanation). The helicase activity was monitored for 600 seconds (Fig 2F). The percent unwinding was plotted against time and the first-order rate constant determined, which was found to be 0.009 sec^-1^ (Fig 2G, a detailed explanation given in S3 Fig).

The reactions performed above were done in the presence of ATP. To confirm that the WCGp80 is an ATP-dependent helicase, the assay was repeated in the presence or absence of either ATP or AMP-PNP (Fig 2H). Moreover, an assay was also carried out with the mutant WCGp80(K201A) in which the essential K residue within the walker A motif was mutated to A (refer, S4 Fig for purification details). Helicase activity was observed only in case of the wild-type protein in the presence of ATP, but not AMP-PNP (Fig 2H). The mutant WCGp80(K201A) did not support helicase activity even when ATP was present (Fig 2H). To investigate the polarity of the WCGp80 helicase, substrates lacking either the 3' or 5' arms of the fork were treated with WCGp80. The results show that WCGp80 unwound a 22 bp dsDNA substrate with a 5' 23- nt ssDNA tail, but not the one that had a 3' tail of the same length (Fig 2I). These results indicate that the unwinding activity was strictly dependent on the presence of a 5' ssDNA tail in the fork structure and hence, WCGp80 is a 5΄→3΄helicase. However, even in the case of the 5' 23- nt ssDNA tailed fork, the overall efficiency appeared low as compared to the case where both tails were present (Fig 2F and 2H). This indicates that while the 5' arm is necessary, it is not sufficient.

Next, substrate saturation kinetic assays were performed using a fluorescence-based method. WCGp80 helicase activity was tested under different substrate concentrations using 20 nM of the enzyme. The initial velocities derived from the dose-response curve (Fig 2J) were plotted against substrate concentrations. The data points thus, obtained were fitted to an allosteric sigmoidal equation. The Hill coefficient was calculated to be 1.6 (Fig 2J and 2K). Protein concentration dependence of helicase activity was also analyzed. Keeping the substrate concentration at saturating level (80 nM, refer Fig 2K), WCGp80 concentration was varied from 5 to 100 nM (Fig 2L). A plot of V_max_ versus protein concentration showed a linear dependence of V_max_ on the protein concentration (Fig 2M). The K_cat_ as derived from the slope of the curve was found to be about 0.007 sec^-1^ which is in reasonable agreement with the first- order rate constant of about 0.009 sec^-1^ obtained in the single turnover kinetic experiment (refer Fig 2G).

### ATPase activity of WCGp80

**Fig 3:**
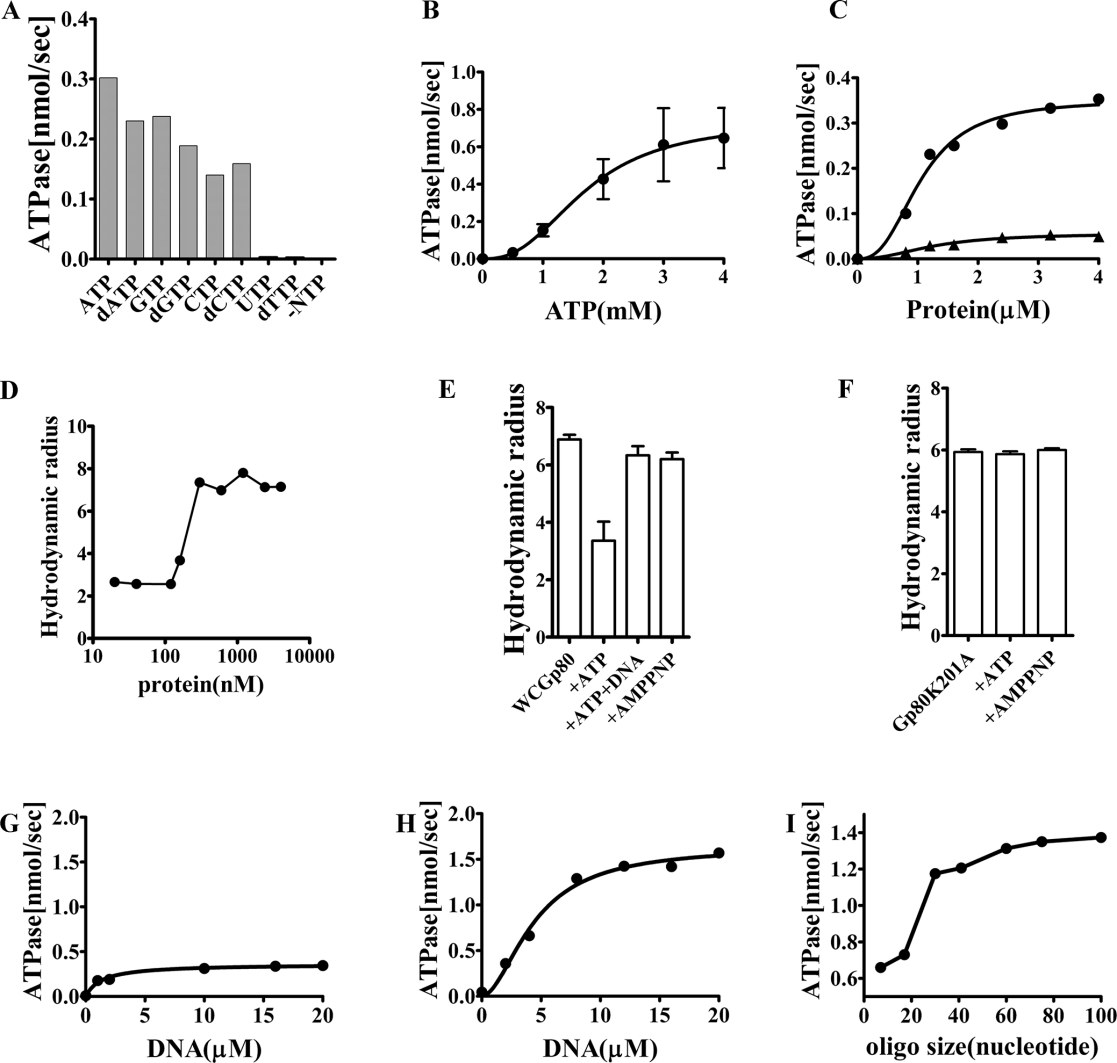
ATPase activity of WCGp80. (A) NTPase activity of WCGp80 using a variety of NTPs as indicated. The assays were performed in the presence of ssDNA (a 41 mer oligonucleotide, S1 Table) at a saturating concentration of 24 μM. The NTPs (or dNTPs as the case may be) were used at a concentration of 4 mM, which was found to be saturating with respect to ATP. (B) Dependence of the ATPase activity on ATP concentration in the presence of saturating concentration (24 μM) of ssDNA, in the form of the 41 mer oligonucleotide. ATP hydrolysis was assayed at the indicated ATP concentrations using the malachite- green method. For each concentration of ATP, reactions were setup in triplicate. The average values obtained ± standard deviations were plotted against ATP concentration. The solid line is the best fit of the data to an equation representing cooperative binding, Y = Ymax*X^h/(K'^h + X^h) which is essentially the same as used to analyze helicase activity (refer Fig 2). **(C)** Enzyme concentration dependence of ATPase activity in absence (triangles) or presence (circles) of 24 μM of 41 mer oligonucleotide. ATP concentration used was 4 mM. ATPase activities were determined at different protein concentrations and plotted. Curve fitting was done using an equation representing cooperative binding which was essentially similar to that used in **(B)**. **(D)** Dynamic light scattering experiments (DLS) performed to estimate the size of WCGp80 at various protein concentrations. The weight averaged hydrodynamic radius (refer S5 Fig) was plotted against protein concentration. **(E and F),** Effect of ATP (4mM), AMP-PNP (4 mM) and DNA (the 41 mer oligonucleotide, 24μM) on the oligomerization status of WCGp80 or its K201A mutant. The combination of cofactors used is shown below. The protein with or without co-factor was incubated for 5 minutes at 25°C prior to analysis. Each experiment was performed thrice. The weight average hydrodynamic radii obtained from individual experiments was again averaged and plotted as mean ± standard deviation. The results of one representative experiment are given in the supplemental information (S5 Fig). **(G-I)** DNA concentration dependent stimulation of ATPase activity measured using oligonucleotides of varying lengths, 17 and 41 mer **(G)** and **(H)** respectively. Curve fitting was done using an equation representing cooperative interactions as mentioned above. **(I)** same experiment was also repeated using oligonucleotides having a broader range of size. In all cases, the oligonucleotide concentration used was 24μM. The maximal ATPase activities obtained were plotted against oligonucleotide sizes. The concentration of WCGp80 used was 2 μM in case of (B), (G), (H) and (I).

DnaB helicases are known to possess intrinsic NTPase activities which are further stimulated by the presence of ssDNA. To test whether WCGp80 possesses this basic attribute, NTPase assays were done in the presence of a 41 mer oligonucleotide (S1 Table). The results show that WCGp80 hydrolyzed several NTPs and also dNTPs. The maximum activity was, however, obtained with ATP (Fig 3A). UTP and dTTP were not hydrolyzed. Substrate saturation experiments were then performed using increasing concentration of ATP (Fig 3B), in the presence of saturating concentration of the 41 mer oligonucleotide (24 μM). The reasons for assuming that this concentration is saturating are mentioned below.

ATP hydrolysis was found to increase in a sigmoidal manner (Hill coefficient 2.3) with the increase in ATP concentration reaching a maximum at 4 mM (Fig 3B). Clearly, there are subunit-subunit interactions as indicated by the sigmoidal nature of the curve. The existence of inter-subunit interactions was also inferred from the protein concentration dependence experiment (Fig 3C). This experiment revealed that the WCGp80 can catalyse the hydrolysis of ATP at a low level. However, in the presence of DNA the activity increases at least 20 fold. Both the basal and DNA stimulated activities were found to increase in a sigmoidal manner (Hill coefficient 2.6) as protein concentration increased.

Dynamic light scattering (DLS) experiments were then performed with WCGp80 to determine its oligomeric status. Such experiments revealed that in dilute solutions the protein exists in a form that has an average hydrodynamic radius of about 3 nm, which in all possibility corresponds to a monomer (Fig 3D, S5 Fig). However, as the concentration increases the radius increases to about 7 nm which corresponds to a hexamer. Oligomerization, however, can be reversed through the addition of 4mM ATP (Fig 3E). The reversal depends on the hydrolysis of ATP, as the non—hydrolyzable analog AMP-PNP, did not have any effect. ATP hydrolysis, therefore, appears to trigger conformational changes in the subunits, the net result of which is the conversion of multimers to monomers. The oligomeric structure was therefore found to be a dynamic one. In the presence of ssDNA (the 41 mer oligonucleotide), however, the oligomeric structure remains stable even though ATP is present. This indicates that externally added DNA stabilizes the oligomeric structure. The WCGp80(K201A) mutant also was oligomeric, but its oligomeric state was not affected by the addition of ATP (Fig 3F). The observation underlines the role played by ATP binding and hydrolysis in the conversion of multimeric to monomeric forms.

DNA-dependent ATPase activity was then measured keeping the ATP concentration at a saturating level (4 mM) (Fig 3G and 3H) and increasing the concentration of oligonucleotides in a stepwise manner. The results indicate that while a 17 mer oligonucleotide stimulated ATPase activity only marginally (Fig 3G), the 41 mer did so significantly (Fig 3H). Stimulation by 41 mer happened in a cooperative manner, and the Hill coefficient was found to be 2. Maximum level activity was obtained at a concentration of about 24 μM. In all the experiments done to assess ATPase activity in the presence of ssDNA, this saturating concentration was used. That this concentration was indeed saturating was evident from not only the ATPase assay experiment (Fig 3H) but also from the direct estimation of binding affinities using fluorescence quenching methods. Such direct methods revealed that the affinity constants (K_d_) of WCGp80 for the 41 mer oligonucleotide, and another oligonucleotide 60 mer in length, were 58 and 164 nM respectively (S6 Fig). The concentration of 24 μM is at least 100 times the K_d_ if not more, which further justifies the argument that this concentration is a saturating one.

In order to assess the effect of DNA length on ATPase activity, the assay was performed at saturating concentration of the oligonucleotides (24 μM) of different lengths. A series of oligonucleotides was chosen from a panel that was already available in the laboratory. These oligonucleotides covering a wide range of lengths from 7 to 100 nts were synthesized earlier for being used to create fork structures such as the one used in the helicase assay mentioned above. The results indicate that the ATPase activity at the saturating ligand concentration varied with the length of the oligo (Fig 3I). However, the variation was not linear. A sharp transition was observed as the length increased from 17 to 30 nucleotides. Beyond 30 nucleotides increase was observed, though less marked. A stretch of 30 nucleotides, therefore, appears to be the minimum length of ssDNA necessary for optimal ATPase activity. However, the maximal activity was obtained when the size of the oligo was in the range of 60–70.

### Stoichiometry of DNA binding by WCGp80

**Fig 4:**
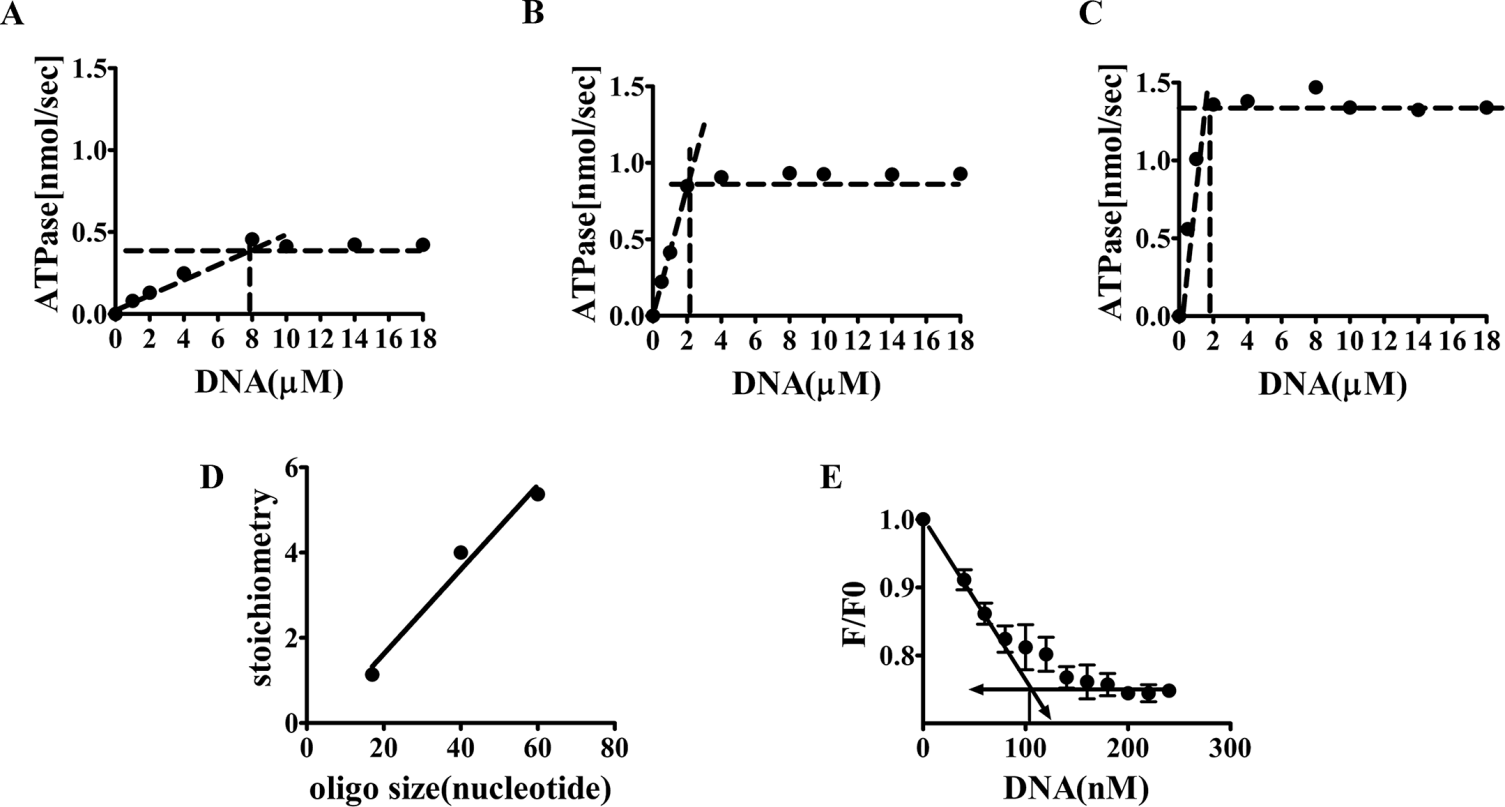
Determination of DNA binding stoichiometries. **(A-C).** Determination of stoichiometry of oligonucleotide binding to WCGp80, using oligomers of different sizes 17, 41, 60. ATPase assays as in Fig 3 were performed with a higher concentration (8 μM) of the enzyme resulting in titration curves. The junction between the lines drawn through the rising and horizontal section is the point where equivalence is reached. **(D),** The estimated number of subunits bound to the oligonucleotides was plotted against their sizes. The best- fit straight line through the points is shown. **(E),** Stoichiometric titrations using the 41 mer oligonucleotide. Fluorescence quenching experiments were performed to determine DNA binding by the protein, WCGp80. In this experiment, the protein concentration used was 400 nM. The junction between the straight lines drawn, through the rising and horizontal parts of the graphs indicates the breakpoint.

To investigate the number of helicase subunits bound per unit length of the oligonucleotide, stoichiometric experiments using the ATPase assay were done. The assumption is that when the protein (receptor) concentration is increased (or K_d_ is decreased), the ligand will have little opportunity to dissociate. In such a situation, the binding will increase linearly until all the receptors get bound. Interaction of DNA with the helicase was assessed by monitoring the resulting increase in ATPase activity. When this experiment was performed, it was found that in the case where a 41 mer oligonucleotide was used, the breakpoint was reached at a DNA concentration that was about three times less compared to that for the 17 mer (Fig 4A and 4B). Similarly when the 60 mer was used, the break happened at an even lower concentration (Fig 4C). Assuming that more than 80% of the protein was active, an attempt was made to estimate the stoichiometries of WCGp80 binding to the ssDNA templates having various sizes. The results showed that the 17 mer ssDNA bound one monomer, whereas the 40 and 60 mers bound 4 and 6 respectively. The data points can be fitted, almost perfectly, to a straight-line (Fig 4D), indicating that the number of subunits interacting with the DNA increased as a linear function of DNA size. From the slope, it is evident that the number of monomers interacting with the DNA increases by one for every 10 nucleotides. In other words, as we go from 17 to 60, the number of monomers that bind increases from 1 to 6 at the rate of one monomer for every 10 nucleotides steps. Thus, if an oligonucleotide is for example, 40 nt in length, it should bind four monomers.

Stoichiometric investigations were also performed using fluorescence quenching experiments [42]. WCGp80, like several other DnaB helicases, possess Tryptophanyl residues within the RecA core region. Addition of oligonucleotides to WCGp80 resulted in quenching of fluorescence in a dose- dependent manner (S6 Fig). As mentioned above, the K_d_ values for the 41 and 60 mer oligonucleotides, 58 and 164 nM respectively were derived based on the quenching phenomenon. The experiments for determining K_d_ were done using a concentration of 40 nM (S5 Fig) which is very close to or lower than the K_d_ values, either 58 or 164 nM. A condition where the receptor concentration is ≤ K_d,_ is ideally suited for the determination of K_d_ but not for stoichiometry. For the latter the receptor concentration must be much higher than K_d_. As the K_d_ of the 41 mer for WCGp80, is about 3 times less compared to that of the 60 mer, therefore it was possible to conveniently achieve a high receptor to K_d_ ratio in the case of the former (41 mer) but not the latter, (60 mer). The stoichiometric determination was thus, performed using the 41 mer. The concentration of WCGp80 used was 400 nM instead of 40 nM to ensure that receptor to K_d_ was sufficiently high. The results of the titration revealed a breakpoint corresponding to a concentration of 100 nM of the oligonucleotide (Fig 4E). This indicated that 4 monomers bind the 41 mer oligonucleotide, an observation consistent with the conclusions drawn from the ATPase assays.

### ATPase activity of the RecA core of WCGp80

**Fig 5:**
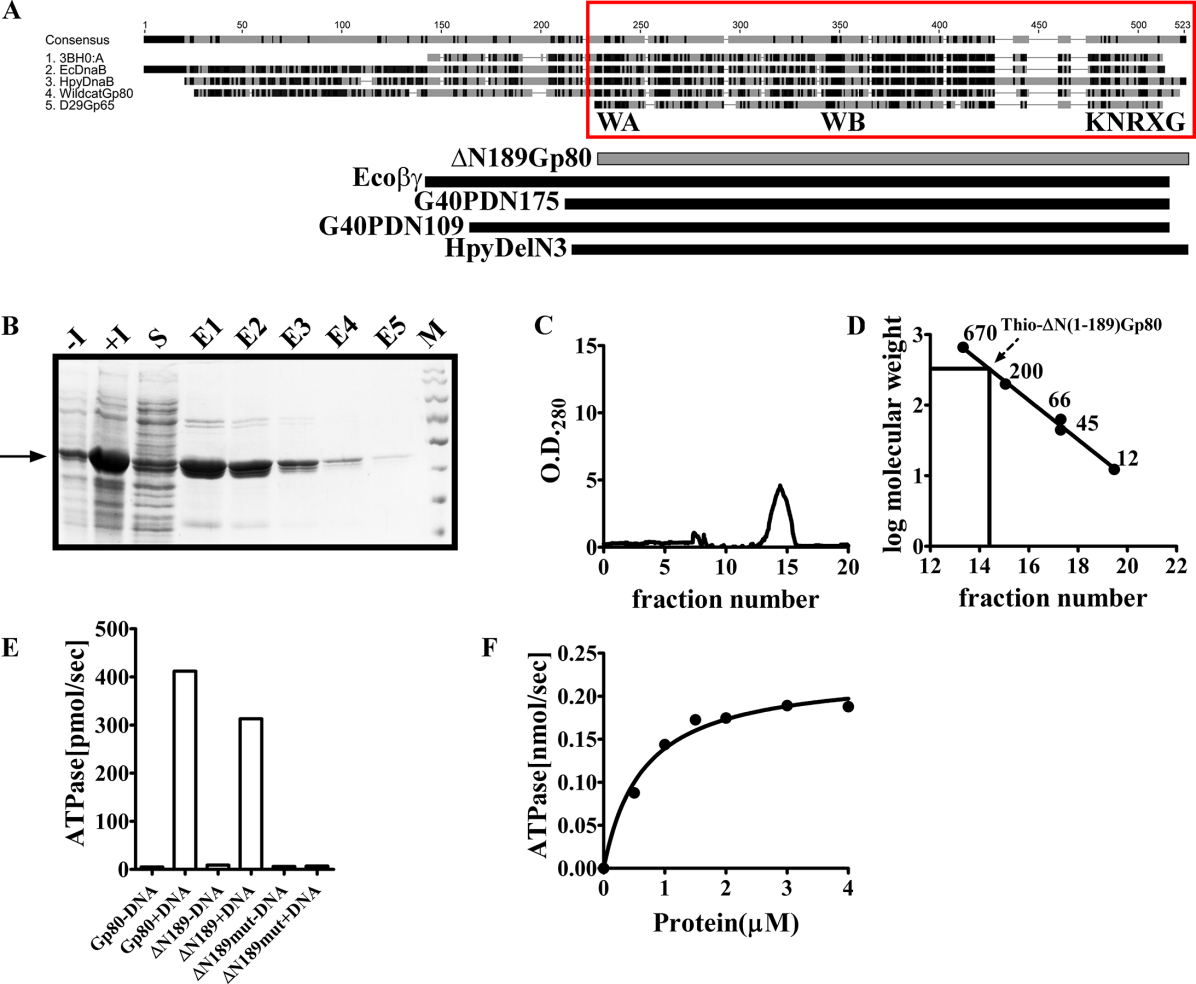
Purification and biochemical analysis of Thio-ΔN(1–189)WCGp80. (A) WCGp80 RecA core as identified by sequence alignment with other DnaB helicase family proteins. The boxed region represents the RecA core as defined in the study. Domain comparison of WCGp80 RecA core (shaded bar) with previously characterized DnaB truncated versions (black bar) like *E*.*coli* βγ fragment (Ecoβγ), *Bacillus* phage G40P truncated versions (G40PDN109 and G40PDN175) and *Helicobacter pylori* DnaB ATPase core (HpyDelN3) is shown schematically below the alignment. (B) A 12% SDS-PAGE analysis of the affinity purified recombinant Thio-ΔN(1–189)WCGp80 protein. The lanes marked–I (before induction), +I (after induction), S (soluble fraction) and E1-E5 represent elution fractions respectively. (C) Size exclusion chromatography to determine the oligomeric status of Thio-ΔN(1–189)WCGp80. The fractions containing proteins after Ni-NTA purification were pooled and passed through a high-resolution Superose 6 size exclusion column. Mw determination (D) was done as described for WCGp80 (Fig 2D and 2E) (E) Comparison of ATPase activity of Thio-ΔN(1–189)WCGp80 (indicated as ΔN189), with those of WCGp80 (indicated as Gp80) and Thio-ΔN(1–189)WCGp80(K201A) (indicated as ΔN189mut) respectively, in the presence (+) or absence (-) of ss-DNA (24 μM, 41 mer oligonucleotide). The protein concentration and ATP concentrations used were 2 μM and 4 mM respectively. (F) Dependence of Thio-ΔN(1–189)WCGp80 ATPase activity on protein concentration in the presence of 24 μM 41 mer oligonucleotide. The solid hyperbolic line represents the best- fit line through the data points.

To obtain a precise definition of what could be considered as the RecA core domain, WCGp80 full- length sequence was aligned with several other DnaB helicase family proteins such as the *E*. *coli* DnaB and the *Bacillus subtilis* phage SPP1 G40P (Fig 5A). Information regarding the domain structure of these two proteins is well known [18, 29]. Also in case of G40P, the crystal structure is available [43]. In addition to these, the single domain protein D29Gp65 characterized in an earlier study [12] was also included. The sequences were aligned and reproduced as a structure-based alignment (S7 Fig) [44]. The region defined as RecA core in this study corresponds precisely to the D29Gp65 sequence (Fig 5A boxed region). It starts immediately upstream of the Walker A motif and continues up to the C- terminus. The core region, thus defined lacks both the N-terminal domain and the linker region as opposed to the βγ domain of *E*. *coli* DnaB helicase, which lacks the N-terminal region but retains the linker region and supports ATPase but not helicase activity[18].

One of the objectives of this study is to investigate the degree to which the RecA core region of WCGp80 can support the functions associated with the full- length protein. The basic function associated with the core of DnaB helicase is the NTPase as mentioned above. Hence, to examine the functionality of the core, it was necessary to first assess the degree to which the domain supports ATPase activity. To address the issue, the RecA core region was synthesized with a Thioredoxin tag at the N-terminal (Thio-ΔN(1–189)WCGp80) in *E*. *coli* and purified to homogeneity using affinity chromatography (Fig 5B). High-resolution SEC performed using the tagged protein revealed that, as in case of WCGp80, it also exists as multimer in solution (Fig 5C and 5D). Unlike in the case of WCGp80, a sufficiently concentrated protein preparation could not be obtained after performing preparative SEC and, therefore, an affinity purified fraction E3, which was substantially pure was used to perform ATPase assays. The results showed that the core region of WCGp80 possessed DNA-dependent ATPase activity comparable to the full-length protein (Fig 5E). The dependence of ATPase activity on protein concentration was then assessed. Instead of a sigmoidal response as observed for the full-length protein (refer Fig 3C), a hyperbolic one was observed (Fig 5F). Overall, the results obtained indicate that unlike the full length protein, in case of the core, cooperative interactions between subunits do not occur.

### DNA helicase activity of Thio-ΔN(1–189)WCGp80

**Fig 6:**
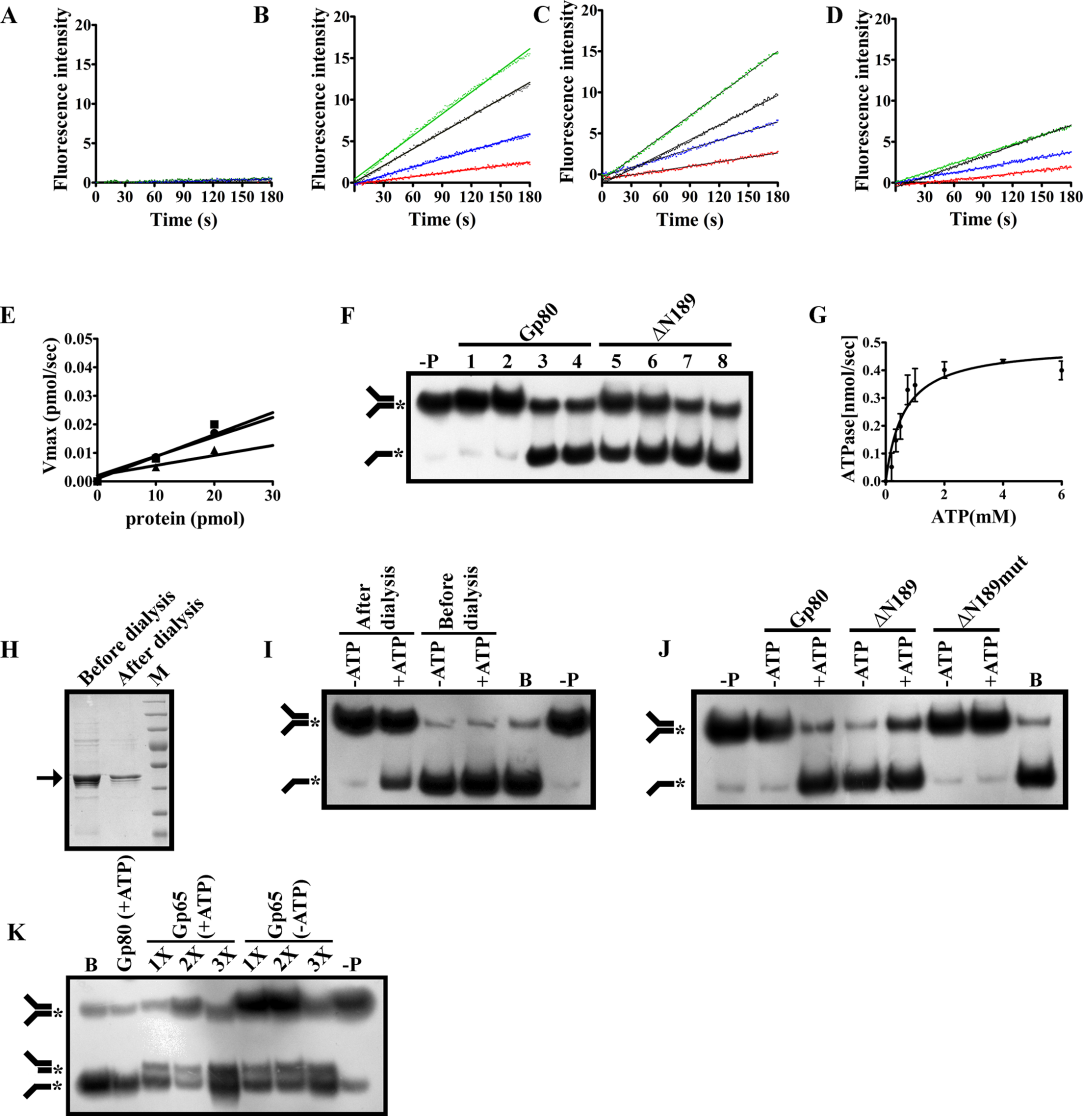
DNA helicase activity of the Thio-ΔN(1–189)WCGp80. Time course of helicase reaction, as assessed by the fluorescence based assays, using different protein concentrations (5,10,20,30 pmole represented by red, blue, black and green lines respectively) of either WCGp80 **(A and B)** or Thio-ΔN(1–189)WCGp80 **(C and D),** with **(B and D)** or without **(A and D)** 4mM ATP**,** keeping the substrate, the fluorescence labeled fork structure, at saturating level (80 nM, refer Fig 2K). Reactions were monitored up to 180 seconds. The V_max_ at each protein concentration was determined from the slope of the linear portion of the graphs. **(E)** Plots of V_max_ versus protein concentrations. The slope of the lines connecting the points, represented by circles, WCGp80 (+ATP); squares, Thio-ΔN(1–189)WCGp80 (-ATP) and triangles Thio-ΔN(1–189)WCGp80 (+ATP) gives the K_cat_. The K_cat_ of WCGp80 and Thio-ΔN(1–189)WCGp80 derivative of WCGp80 in the presence of ATP were 0.00154 sec^-1^ and 0.0007 sec^-1^ respectively while that of Thio-ΔN(1–189)WCGp80 in the absence of ATP was 0.001361 sec ^-1^. **(F)** Comparison of the helicase activity of WCGp80 (lanes 1 to 4) with Thio-ΔN (1–189)WCGp80 (lanes 5 to 8) by radiometric helicase assay in the absence (lanes 1, 2 and 5, 6) and presence (lanes 3, 4 and 7, 8) of ATP (4mM) at two different time points, 5min (lanes 1, 3, 5 and 7) and 10mins (lanes 2, 4, 6 and 8). Lane (–P) indicates absence of protein. **(G)** Substrate saturation experiment performed to evaluate the affinity of Thio-ΔN(1–189)WCGp80 for ATP. ATPase activities of the protein at various ATP concentrations were determined. Each determination was done in triplicate. The ATPase activity (mean ± standard deviation) was plotted against substrate concentration. Curve fitting was done using a Michelis-Menten type equation Y = Ymax*X/(Kd + X), where K_d_ is the half maximal velocity, Y the ATPase activity at a particular protein concentration (X) and Ymax the activity at infinite substrate concentration. **(H)** 12% SDS-PAGE analysis of Thio-ΔN(1–189)WCGp80 before and after extensive dialysis to remove bound NTPs. The position of the band corresponding to the desired band is indicated by an arrow. **(I)** Effect of dialysis on ATP dependence of helicase activity of Thio-ΔN(1–189)WCGp80. The assays were carried out with the samples (before and after extended dialysis respectively) in the presence or absence of ATP (- or +). B and (-P) refer to boiled substrate and without protein respectively. **(J)** An experiment similar to (I) was done with WCGp80 (indicated as Gp80), Thio-ΔN(1–189)WCGp80, (indicated as ΔN189) and its mutated version Thio-ΔN(1–189)WCGp80(K201A) (indicated as ΔN189mut) in the presence or absence of ATP. **(K)** Assay for the structure specific nuclease activity of D29Gp65 (indicated as Gp65) purified as described earlier [12] (S9 Fig) in the presence or absence of ATP (- or + as indicated) and increasing concentrations of the protein. 1X corresponds to 220 nM. A control lane showing ATP dependent helicase activity mediated by WCGp80 (Gp80+ATP) was also incorporated.

To this point, we were able to demonstrate that the RecA core domain of WCGp80 fused to a Thioredoxin tag is capable of performing DNA dependent hydrolysis of ATP. It was of considerable interest to determine whether the energy from ATP hydrolysis could be used for helicase activity. Hence, helicase activities of WCGp80 (Fig 6A and 6B) and Thio-ΔN(1–189)WCGp80 (Fig 6C and 6D) were assessed using a fluorescence based assay in which the substrate concentration was kept at the saturating level (80 nM). In the absence of ATP (Fig 6A) no significant unwinding activity was observed in case of WCGp80. However, in the presence of ATP (Fig 6B), time dependent DNA unwinding by WCGp80 was found to occur as expected (Fig 6B). The response was linear, and the slope of each line represents V_max_ for a given concentration of the enzyme. With increasing concentrations of the protein, V_max_ was found to increase linearly (Fig 6E, circles). Intriguingly, in case of Thio-ΔN (1–189)WCGp80, helicase activity was observed, even in the absence of ATP (Fig 6C). In fact, addition of ATP had a negative effect (Fig 6D). The slope of the V_max_ against protein concentration curve which is a reflection of the turnover number was almost same between WCGp80 in the presence of ATP and Thio-ΔN(1–189)WCGp80 in its absence (Fig 5E, circles and squares respectively). The results indicate that the core region in itself is capable of generating helicase activity, although surprisingly the function appeared to be ATP independent.

DNA unwinding was further monitored qualitatively using a radiometric assay. As is evident from the autoradiogram, DNA unwinding by WCGp80 was strictly dependent on the presence of ATP (Fig 6F, lanes 3 and 4 compared to 1 and 2). In case of Thio-ΔN(1–189)WCGp80, however, helicase activity was discernible not only in the presence but also in the absence of ATP (Fig 5F, lanes 7 and 8 compared to lanes 5 and 6). The ability of Thio-ΔN(1–189)WCGp80 to function apparently in an ATP-independent manner was an unusual observation. One possibility could be that this derivative has higher affinity for ATP than the full length protein and thus the purified protein contained substantial amount of pre-bound ATP. To examine this possibility several investigations were done. First to determine the K_m_ for ATP, ATPase assays were performed at various ATP concentrations. The results (Fig 6G) indicated that, unlike in the case of the wild type protein (Fig 3B), substrate saturation curve for ATP in case of Thio-ΔN(1–189)WCGp80 derivative was hyperbolic. Moreover, the concentration at which half maximal velocity was achieved was 3 times less in case of Thio-ΔN(1–189)WCGp80 as compared to the wild type protein (0.5 mM, Fig 6G as compared to 1.7 mM, Fig 3B). The results obtained suggest that indeed Thio-ΔN(1–189)WCGp80 has a higher affinity for ATP and for this reason the isolated protein was already saturated with ATP (or any other NTP). To further investigate this, the OD _260_/ OD_280_ ratios of the Thio-ΔN(1–189)WCGp80 and WCGp80 protein samples were measured. The ratio was found to be 1.08 in case of Thio-ΔN(1–189)WCGp80 but only 0.59 for WCGp80. This indicated further that the observed ATP independent activity was perhaps a manifestation of the fact that the purified protein was pre-saturated with NTP. To prove this point the protein was subjected to another round of dialysis, a method used in our previous study, to get rid of pre-bound NTP [12]. Following dialysis the OD _260_/ OD_280_ ratio went down to 0.6, an indication that the bound NTP was eliminated at least partly if not completely. The dialyzed sample was resolved on a SDS-PAGE to confirm that the protein was not lost during the process (Fig 6H). The dialyzed sample when assayed for helicase activity was found to function in an ATP dependent manner (Fig 6I, after dialysis compared to before). To finally confirm that the activities observed are due to Thio-ΔN(1–189)WCGp80, and not any contaminant, the activity of a mutant version, Thio-ΔN(1–189)WCGp80(K201A) was examined. The mutant version which carried a K to A mutation in the Walker A motif was inactive (Fig 6J) confirming the specificity of the observations. Interestingly, D29Gp65 which was earlier demonstrated to be a fork specific nuclease also possessed similar ATP independent properties as reported earlier [12]. To emphasize this similarity further, the D29Gp65 mediated nuclease assay performed in the previous study was redone and the results presented (Fig 6K). The inherent similarity in how Thio-ΔN(1–189)WCGp80 and D29Gp65 function is worth noting. Although the end results are somewhat different, in one case it is truncation and the other unwinding, both act on fork structures, and interestingly, their ATP dependence properties appear to be similar.

## Discussion

Several Mycobacteriophage genomes encode homologs of DnaB helicases. Many of these, such as D29Gp65, however, are truncated versions comprising only the RecA core region and devoid of the N-terminal domain. The function of these single domain RecA core proteins remains enigmatic. Phylogenetic analysis of the RecA superfamily proteins performed in this study, and also previously, indicate that all the members of the family possess a common core corresponding to the structural element known as RecA fold and thus they may have diverged from a common ancestor [14]. According to the hypothesis put forward in this investigation, the last universal common ancestor of the RecA superfamily must have been a single domain protein. The existence of single domain RecA core proteins, such as the archaeal recombination related proteins RadB and aRadC (referred to as ‘mementos from the last universal common ancestor’ in another study [33]) and the mycobacteriophage encoded D29Gp65 homologs, in the extant world supports such a possibility. The hypothesis originating from phylogenetic analyses however, requires experimental validation, which is what we attempted in this study. The model system used is WCGp80, a DnaB helicase family protein encoded by the mycobacteriophage Wildcat. The reasons behind choosing this protein are various. First of all, it roots deep in the DnaB helicase family indicating that the protein may be an extant version of an ancient DnaB type protein. Second, it shares considerable homology with the D29Gp65 class of single domain proteins derived from mycobacteriophages. The phylogenetic tree constructed indicates that D29Gp65 like single domain proteins may have been the ancestors of the DnaB family of which WCGp80 happens to be a deep rooting member. Considering that D29Gp65, despite having the minimal core domain possesses a specific biochemical function [12], it was considered worth testing, whether WCGp80 core domain which shares considerable homology with D29Gp65 also possesses the same or a similar activity. If this activity can be characterized then it may be possible to garner necessary experimental support to the proposal that the full-length proteins in the RecA family in general, and DnaB, helicases in particular, evolved from single domain proteins that existed in the evolutionary past, which were themselves functional.

To find an answer to the questions raised above, the biochemical characterization of the full- length protein WCGp80 was attempted as no information about this protein was available in the literature at the time when this investigation was initiated. The purified protein was found to exist as a hexamer. The hexamer, was however, unstable and at lower concentrations it dissociated to give rise to monomers, as was evident from DLS experiments. Previous investigations have shown that *E*. *coli* DnaB helicases, exist as multimers over a broad concentration range from 100nM- 10µM [39]. The results present in this study show that if the concentration is decreased below 300 nM, the oligomeric structure breaks down. Hence, in case of this protein, the hexameric structure appears to be more unstable as compared to that of *E*. *coli* DnaB. Incubation in the presence of ATP also resulted in destabilization of the multimer. For the transition to occur, ATP hydrolysis was found to be necessary as a nonhydrolyzable analogue did not support the conversion. The addition of single-stranded DNA, however, stabilized higher oligomers in the presence of ATP. The ability of WCGp80 to transit between the two forms, multimeric and monomeric possibly allows it to load onto DNA as has been proposed earlier [45].

WCGp80 possesses a low level of intrinsic ATPase activity, which can be stimulated in the presence of ssDNA. ATP is, however, not the only nucleotide triphosphate that the protein can hydrolyze. Other nucleotide triphosphates including those corresponding to the deoxynucleotides were also hydrolyzed. ATP nevertheless, appeared to be a better substrate compared to others. Although the protein hydrolyzed several NTPs, including dNTPs, it failed to utilize dTTP which is known to be optimally used by the T7 helicase [46]. All the activities of WCGp80 were found to be cooperative in nature. In this aspect, WCGp80 resembles T4Gp41, which is known to hydrolyze GTP in a cooperative manner, particularly in the presence of ssDNA [31]. In hexameric helicases which include the T4 and T7 helicases, NTP hydrolysis is necessary for movement along the ssDNA. The mechanism by which hydrolysis leads to movement however remains unclear, but nevertheless several models have been proposed, including a staircase mechanism in which the hexamer forms a nonplanar staircase structure, through which the ssDNA migrates either in the 3'- 5' direction or its opposite [47, 48]. In general it has been suggested that the subunits of the hexamer are nonindentical with respect to their ability to bind and hydrolyze NTPs. Thus even though there are six possible sites for ATP and DNA, only three develop a high affinity for these cofactors at a given point of time [49]. Positive cooperativity has been found to exist between these three high-affinity sites. The degree of cooperativity in case of WCGp80, as evident from the Hill Coefficients, was found to be close to 3 indicating that as in case of other hexameric helicases three monomers are involved in the cooperative interactions at any instant in case of WCGp80.

The binding stoichiometry was determined assuming that the protein hydrolyzes ATP, only if it is bound to the DNA. This assumption is based on the Scheme I proposed in an earlier study [50]. The stoichiometry experiments were done with a highly purified preparation of the enzyme obtained after performing SEC. However, there is no way to know the exact fraction of the protein present in the sample that is active. As a thumb rule, one can assume this to be 80%. The conclusion that a 17 mer binds to a monomer will not change significantly if about 20% is considered inactive. A more precise idea about the stoichiometry can be obtained from the observation that the number of bound monomers increased linearly with DNA size, and the rate was found to be one per 10 nucleotide steps. This explains why the 17 mer accommodated only one monomer. Apparently, monomer binding is quantized and takes place in steps involving 10 nucleotides. The stoichiometry deduced based on ATPase assays was further confirmed by performing a fluorescence quenching experiment with the 41 mer oligonucleotide. It differs from that estimated for the T7 Gp4 helicase which was found to be six monomers (hexamer) for an oligonucleotide having a size of 10–30 nucleotides and twelve for an oligo of 40–60 nt in size [51]. Interestingly in case of T4Gp41, the stoichiometry, was found to be one for an oligo of around 20 nucleotides [52]. The observed differences in the binding stoichiometries possibly is a reflection of the differences in the manner in which multimers are formed on the ssDNA. In the case of T7 helicase (Gp4), a hexameric ring is formed around the ssDNA which explains why the number of monomers that are engaged by a short stretch of DNA, only 20 nucleotides long, is as high as 6 [51]. In the case of WCGp80 the arrangement could be different, possibly similar to that of the Rho helicase, where the RNA contacts every subunit of the hexamer [53].

An important observation made in this study is that V_max_ of DNA-dependent ATPase activity increased with oligo length. It increased four-fold as the length increased from 17 to 30 nucleotides. This is a general feature of many helicases. However, after this sharp rise no further change was observed, at least not significantly. The results obtained are similar to that obtained in a previous study using T4Gp41 where a rapid increase was observed from 0–20 nucleotides, but with oligos larger than 20 nucleotides, the V_max_ increased only marginally [52]. The reason why the 17 mer is unable to activate the ATPase activity of WCGp80 substantially is possibly because in this case, a multimeric unit cannot be assembled. An alternative explanation could be that the helicase has very little opportunity to translocate along the DNA, when it binds the 17-mer oligonucleotide but it can do so when it interacts with the longer template. As explained earlier, [31] longer the helicase stays on the DNA, as is the case when translocation occurs, more will be the ATPase activity.

An important revelation of this study is that the central RecA core itself has the ability to support helicase activity. Ideally, it would have been appropriate to use the core itself without any fusion at the N-terminal end. Constructs were indeed made to overproduce the core only. Although the synthesis of the protein takes place from such constructs, no recovery was possible in the soluble fraction (S8 Fig). Instability of the core domain alone has been reported in a previous study too [29]. Most of the detailed investigations with the core were done using *E*. *coli* DnaB helicase. Based on domain analysis, several functional fragments of this helicase viz. α, β and γ have been described [18]. The α and γ fragments represent the N and C-terminal domains, and β, the central core spanning Walker A, but not the B, motif. The β fragment was sufficient to support ATPase activity at a minimal level. In DnaB helicases, the N-terminal domain is linked to the core through a linker. In the βγ fragment, the N-terminal domain is missing, but the linker domain is still present. This fragment failed to support helicase activity indicating an indispensable role for the N-terminal end. The N-terminal domain deletion mutant of WCGp80 examined in this study lacks not only the N-terminal one but also the linker region. It was, therefore, surprising to see that this core region possesses substantial helicase activity. The failure to associate activity with the central fragment in earlier studies is probably because the core is highly unstable and denatures easily as has been proposed for the *Bacillus* phage helicase G40P [29]. Hence, the additional feature introduced in this study–a thioredoxin tag, possibly induced stability and improved protein folding. Thus, the canonical N-terminal region of DnaB helicases is not absolutely necessary. Indeed, pRSF1010 RepA, which possesses a much shorter N-terminal region as compared to DnaB [36] is fully functional. However, deletion of a few amino acid residues from the N-terminal end of this protein abolishes activity. It has been suggested that the N-terminal region of RepA serves as a hook, which is essential for hexamerization [37]. The primary function of N-terminal domains may thus be to aid multimerization. It is possible that the N-terminal thioredoxin tag may do the same either directly by acting as a hook or indirectly by promoting the proper folding of the protein. Although the presence of N-terminal domain could help in bringing about multimerization, its requirement doesn’t appear to be absolute. Proteins such as KaiC which lack N-terminal domains, can multimerize indicating that the core itself has sufficient information embedded in its primary sequence that may be considered necessary and sufficient for multimer formation [19]. Whatever the case may be; the results show that the canonical N-terminal region of DnaB helicase is dispensable for both helicase and ATPase functions and can be replaced by unrelated polypeptide sequences. However, the presence of the N-terminal domain helps in maintaining cooperativity between subunits as is evident from the observation that the substrate saturation curves for ATPase were hyperbolic in case of the Thio- tagged core domain polypeptide, but sigmoidal in the case of the full- length protein. The affinity for ATP is also more in case of the core. These results indicate that the full-length protein will be able to function, only if the ATP concentration crosses a certain value, whereas the truncated protein would remain active even when ATP concentration is low. This is evident from the observation that the core protein isolated from the cell had a higher level of bound ATP as compared to the full-length protein and thus functioned in an apparently ATP-independent manner. Interestingly, D29Gp65 which also lacks a N-terminal domain functions in a similar ATP- independent manner as reported in the previous study [12] and reproduced here. Thus, one major difference between the single domain proteins and their full- length counterparts appears to be in the extent of ATP requirement. The single domain proteins, at least those tested here, function at a lower ATP concentration as compared to the full length ones.

From the evolutionary point of view, the findings presented here are important. They throw more light on the evolution of the RecA family proteins, all of which possess a core that corresponds to the truncated WCGp80 helicase used in this study. The RecA core in itself is capable of generating function at least in some cases. Studies with fragments of RecA protein have revealed that even small peptides derived from the core region can catalyze recombination [54]. Similarly KaiC, the circadian rhythm regulator in cyanobacteria, is a functional protein even though it essentially has only the RecA core domain [55]. Gp65 the mycobacteriophage derived RecA core protein was found to be a nuclease. In archaea, there are several single domain RecA core proteins, which can function as recombinases even though additional domains are absent [15]. Given these instances, it appears that basic functions associated with the members of the RecA family can be supported by the NTPase core itself. The remaining domain(s) merely serve to modify the functional properties. The bottom-up model proposed here appears to contradict a top-down model proposed to explain how DnaB helicases evolved from fused primase-helicases [56]. In the latter model it is assumed that primase helicase fusion proteins evolved first and then subsequently split. While splitting the junction region got duplicated which is why the N-terminal domain of DnaB helicases have sequence similarity with the C-terminal sequence of DnaG, which functions as a primase in eubacteria. In the light of the present study the model may be modified in a minor way by suggesting that intially a RecA core domain which had the ability to unwind DNA or potentially capable of doing so, got fused to a DnaG primase resulting in a fusion protein. This could have happened on a phage as illegitimate recombinations are common in them. Subsequently, the split occurred. However, in the process, the core region gained the N-terminal domain. Given that evolution usually proceeds from simpler to more complex structures (bottom up), such an explanation is likely to be valid.

## Supporting Information

S1 Fig Alignment of core domains of various members of the RecA superfamily including WCGp80. (PDF)

S2 Fig Detailed phylogenetic tree derived from the alignment presented in S1 Fig. (PDF)

S3 Fig Determination of the binding constant (K_d_) for the interaction between WCGp80 and fork DNA substrate. (PDF)

S4 Fig Purification of WCGp80(K201A) by affinity chromatography and size exclusion chromatography using Sephacryl S-200 column. (PDF)

S5 Fig Distribution of hydrodynamic radii of WCGP80 and WCGp80(K201A) in the apo state as well as in the presence and absence of various cofactors. (PDF)

S6 Fig Equilibrium binding of oligonucleotides to WCGp80 studied by intrinsic fluorescence quenching experiment. (PDF)

S7 Fig Structure based alignment of Wildcat Gp80 protein. Flat figure representation of Wildcat Gp80 alignment using ESPript 3.0.with PDB entry 3BH0.
(PDF)

S8 Fig 12% SDS-PAGE analysis of total protein extracted from E. coli cells synthesizing ΔN(1–189)WCGp80 from a recombinant vector based on the expression plasmid pET28a. (PDF)

S9 Fig 12% SDS-PAGE analysis of affinity purified D29Gp65 protein that was used in the structure specific nuclease assays. The WCGp80 and Thio- ΔN(1–189)WCGp80 proteins were run in the same gel as controls.
(PDF)

S1 Table Substrates used in DNA unwinding and ATPase assays. (PDF)
