# Supplementary material for: Biochemical Characterization of a Mycobacteriophage Derived DnaB Ortholog Reveals New Insight into the Evolutionary Origin of DnaB Helicases
Source: PLoS One. 2015 Aug 3;10(8):e0134762. doi: 10.1371/journal.pone.0134762 (PMC4523182; doi:10.1371/journal.pone.0134762)
Supplement: S1 Fig — (PDF) [file pone.0134762.s001.pdf]

```
                   10        20        30        40        50        60        70        80
          ....|....|....|....|....|....|....|....|....|....|....|....|....|....|....|....|
pRSF1010  -------------EAFAAAPPPLDYVLP--NMVAGTVGALVSPGGAGKSMLALQLAAQIAGGPDLLEVG-----ELPTGP
T4        --------------KVPFKLRILNKITK-GGAETGTLNVLMAGVNVGKSLGLCSLAADYLQLG---------------HN
Gst DnaB  -----------------------------------DLIIVAARPSVGKTAFALNIAQNVATKTNEN------------VA
SPP1      ----------------GVPSGFTELDRMTYGYKRRNFVLIAARPSMGKTAFALKQAKNMS-DNDDV------------VN
T7        --------------LLFSGCTGINDKTL--GARGGEVIMVTSGSGMGKSTFVRQQALQWGTAMG-------------KKV
Mge DnaB  ----------------GLSSGFLKLDQLTSGWKPGELIVIAARPGRGKTALLINFMASAAKQIDPKTDV---------VL
Wildcat   -----------------------------------RMLVVGARPGCGKTVFVSQLGFYAALQHLSV-------------L
D29       ------------------------------------MVLVCAGPGTGKSAFVLAYALKSKVPT----------------L
Che12     ----------------PLPDVWDALIQKGTHLRRGQLCLVCAGPGTGKSAFVLAYALKSRVPT----------------L
Msm DnaB  -----------------VPVRCVEVDNPE-----HLYLAGPGMVPTHNSTLGLDFMRSCSIKHRMA------------SV
BxZ2      ------------------------------------LALVCAGPGTGKSAFVLTYALLARVPT----------------L
BxB1      ------------------------------------LALIAAAPGGAKSAFTLALALKGRIPT----------------Y
244       ------------------------------------LVVVGGRPGAGKSNAGLNIISKAAEDKLKS-------------L
Cjw1      ----------------PTPWPELNRWIPGGGFSPGNLVVVGGRPGAGKSNAGLNIISKAAEDKLKS-------------L
Gladiator MYTPRQSLYIRGTAGDPLPTVWQSLSQKGTSLRRGQLVLVCAGPGTGKSAFVLAYALKSKVPT----------------L
Trixie    MYTPRQSLYIRGSAGDPLPTVWNALEMKGTQLRRGQLVLVCAGPGTGKSAFVLAYALKSKVPT----------------L
First     MYTPRQSLYIRGSAGDPLPTVWNAIEMKGTTLRRGQLVLVCAGPGTGKSAFVLAYALKSKVPT----------------L
Redrock   MYTPRQSLYIRGSAGDPLPPVWSALTHKGTTLRRGQLVLVAAGPGTGKSAFVLAYALKSKVPT----------------L
EricB     MYTPRQSLYIRGTAGDPLPTVWQSLSQKGTEFRRGQLVLVCAGPGTGKSAFVLAYALKSKVPA----------------L
Turbido   MYTPRQSLYIRGSAGDPLPPVWNALDMKGTQLRRGQLVLVCAGPGTGKSAFVLAYALKSKVPT----------------L
Msm*      ------------------------------------MIIIAGRPGHGKSTLALDIMRSCSIKHEMA------------SI
Vch RecA  --------------TISTGSLSLDIALGAGGLPMGRIVEIFGPESSGKTTLTLELIAAAQREG---------------KT
Hpy RecA  --------------AISTGSLGLDLALGIGGVPKGRIIEIYGPESSGKTTLSLHIIAECQKNG---------------GV
Sau RecA  --------------TTSTGSVTLDNALGVGGYPKGRIIEIYGPESSGKTTVALHAIAEVQSNG---------------GV
Msm RecA  --------------VIPTGSISLDVALGIGGLPRGRVIEIYGPESSGKTTVALHAVANAQAAG---------------GI
Bam RecA  --------------TVPSGSLALDTALGIGGYPRGRIIEVYGPESSGKTTVALHAIAEVQEKG---------------GQ
Eco RecA  --------------TISTGSLSLDIALGAGGLPMGRIVEIYGPESSGKTTLTLQVIAAAQREG---------------KT
Pvu RecA  --------------TISTGSLSLDIALGAGGLPMGRIVEIYGPESSGKTTLTLQVIAAAQREG---------------KT
Hin RecA  --------------SISTGSLGLDVALGIGGLPMGRIVEIFGPESSGKTTLTLSVIAQAQKAG---------------KT
Osa DCM1  --------------RITTGSQALDELLG-GGIETLCITEAFGEFRSGKTQLAHTLCVSTQLPI---------HMHGGNGK
Hsa DCM1  --------------HITTGSQEFDKLLG-GGIESMAITEAFGEFRTGKTQLSHTLCVTAQLPG---------AGGYPGGK
Ehi DCM1  --------------KITTGSSQFDQLLG-GGIETMSVTEMFGEFRTGKTQLCHTLAVTTQLPS---------HLKGGNGK
Ath DCM1  --------------KITTGCQALDDLLG-GGIETSAITEAFGEFRSGKTQLAHTLCVTTQLPT---------NMKGGNGK
Zma DCM1  --------------RITTGSQALDELLG-GGIETLCITEAFGEFRSGKTQLAHTLCVSTQVPI---------HMHGGNGK
Sce Rad51 --------------CLTTGSKNLDTLLG-GGVETGSITELFGEFRTGKSQLCHTLAVTCQIPL---------DIGGGEGK
Hsa Rad51 --------------QITTGSKELDKLLQ-GGIETGSITEMFGEFRTGKTQICHTLAVTCQLPI---------DRGGGEGK
Tbr Rad51 ---------------------EVDKLLG-GGIEVGSITELFGEFRTGKTQLCHTLCVTCQLPL---------SQGGGEGM
Bmo Rad51 --------------QLTTGSKELDRLLG-GGIETGSITEIFGEFRTGKTQLCHTLAVTCQLPI---------EQSGGEGK
Mmu Rad51 --------------QITTGSKELDKLLQ-GGIETGSITEMFGEFRTGKTQICHTLAVTCQLPI---------DRGGGEGK
Xla Rad51 --------------QIGTGSKELDKLLQ-GGIETGSITEMFGEFRTGKTQLCHTLAVTCQLPI---------DRGGGEGK
Eco Sms   --------------RFSTGFKEFDRVLG-GGVVPGSAILIGGNPGAGKSTLLLQTLCKLAQ-Q---------------MK
Bsu Sms   --------------RVKTQLGEFNRVLG-GGVVKGSLVLIGGDPGIGKSTLLLQVSAQLSGSS---------------NS
Mtu Sms   --------------PCPTGIDELDRVLG-GGIVPGSVTLLAGDPGVGKSTLLLEVAHRWAQSG---------------RR
Ape RadA  -------------------------LLG-GGIETKTITELFGEFGSGKTQICHQLSVNVQLPE---------DKGGLEGK
Hpy Sms   -------------------------VLG-GGIAKGGLYLVGGSPGVGKSTLLLKVASGLAKNQ---------------QK
Tpa Sms   --------------------------LG-GGAVRRSAIMIGGEPGIGKSTLLLQIAAACGK------------------S
Eco DnaB  ----------------GVNTGYDDLNKKTAGLQPSDLIIVAARPSMGKTTFAMNLVENAAMLQDKP------------VL
Bsu DnaB  ----------------GIPTGFTELDRMTAGFQRNDLIIVAARPSVGKTAFALNIAQNVATKTDES------------VA
Mtu DnaB  ----------------GVATGFTELDEVTNGLHPGQMVIVAARPGVGKSTLGLDFMRSCSIRHRMA------------SV
Afu RadA  --------------KITTGSKDLDELLG-GGVETQAITEFFGEFGSGKTQICHQLAVNVQLPE---------DEGGLEGS
Mja RadA  --------------KLSTGSKNLDEILG-GGLESQSVTEFACMFGSGKTQIAHQACVNLQCPERIVADDAIKDEILNEPK
Mth RadA  --------------RITTGSKALDELIG-GGIETQAITEVFGEFGSGKSQLSHELAVTVQLPE---------ERGGLDAE
Sis RadA  --------------KISTGSQALDGLLA-GGIETRTMTEFFGEFGSGKTQLCHQLSVNVQLPP---------EKGGLSGK
Mth RadB  -------------RRIPT-ESSIDRILG-GGVERRTITQFYGPPGSGKTNITIKLAVETARRG---------------KN
Mja RadB  ---------------------MLKEILL-GNAEKGIITQIYGPPGVGKTNICIINSINAVNSG---------------K-
Mma RadB  ---------------------MLEELLN-GNIEKKTITQIYGPPGVGKTNICILSMLKAIENG---------------KN
Afu RadB  --------------LIPTGSKCIDSLLG-GGVETGTVTQIYGHGGTGKTTLCLMLAKNAAEQF---------------K-
Hsa RadB  --------------HLPTGCGALDELLG-GGVERGTVTQLYGPPAAGKTNVALTTAVTTAAAG---------------GL
Pfu RadB  --------------LLTTGVKGLDELLG-GGVAKGVILQVYGPFATGKTTFAMQVGLLNE------------------GK
Hvo RadB  --------------SVSTGCDALDDLLD-GGFERGTVTQVYGPSAAGKTNVALSAAVRVAAAG---------------GT
Tga RadB  --------------MLSTGVKSLDELLG-GGFGEGVLTQIYGPYATGKTTLAVQTGLLSG------------------KK
Mma KaiC  ---------------------ELDRKLS-GGYPGNKATLITGVAGSGKTIYGIHFLHRSCVEG---------------RK
Sel KaiC1 --------------KMRTMIEGFDDISH-GGLPIGRSTLVSGTSGTGKTLFSIQFLYNGIIEF--------------DEP
Sel kaiC2 --------------RVSSGVVRLDEMCG-GGFFKDSIILATGATGTGKTLLVSRFVENACAN---------------KER
Mth KaiC1 --------------KAPTGIKGLDMITE-GGFPRGRNTLIYGGPGTGKTFIAMEFLLKGASVY--------------GEP
Mth KaiC2 --------------LVSTGIPTLDEMLG-GGVYRGSAVLVSGTTGAGKTSLLSKFAYESCRR---------------GER
Sso aRadC --------------RLSTGILDFDKLIQ-GGIPQGFFIALTGEPGTGKTIFSLHFIAKGLRD---------------GDP
Pae aRadC --------------RVRSYVPGLDEILF-GGIPERSVVLLSGGPGTGKSILGKQFLYNGLKR---------------GDA
Pca aRadC --------------RAPTGIWYVDQLLQ-GGFRRGEIYLIAGEAGQGKTIFSLQFLKTGAELYD-------------EP
```

```
Tma aRadC    --------------RVKTGIPGMDEILH-GGIPERNIVLISGGPGTGKTIFSQQFIWNGLQM---------------GEP
Tko aRadC    --------------RVKTGIPGMDEILH-GGIPERNVVLLSGGPGTGKSIFSQQFLWNGLQM---------------GEP
Ape aRadC    --------------RVKTGIPGMDDILY-GGIPRRNVVLLSGGPGTGKSIFSYQYLWNGLRE---------------GEP
Hbu aRadC    --------------RLPTGVPGFDKLIQ-GGIPRGFFVAVVGEPGTGKTIFSIHFIAEGIRQ---------------GDK
Sma aRadC    --------------RIPTGVKGLDEILR-GGIPRGFVVAVVGEPGTGKTVFCIHFIGKGLEM---------------GDK

                      90        100       110       120       130       140       150       160
             ....|....|....|....|....|....|....|....|....|....|....|....|....|....|....|....|
pRSF1010     VIYLPAEDP---PTAIHHRLHALGAHLSAEE---------------RQAVADGLLIQPLIGSLP-------------N
T4           VLYISMEMAEEVCAKRIDANMLDVS------------------------LDDIDDGHISYAEYK--------------
Gst DnaB     IFSLEMSAQ--QLVMRMLCAEGNINAQNLRTG--KLTPEDWGK---LTMAMGSLSNA-GIYIDDT------------PS
SPP1         LHSLEMGKK--ENIKRLIVTAGSINAQKIKAARRDFASEDWGK---LSMAIGEISNS-NINIFDK------------AG
T7           GLAMLEESVE-ETAEDLIGLHNRVRLRQSDSLKREIIENGKFD-----QWFDELFGNDTFHLYDS-----------FAE
Mge DnaB     FFSLEMRNR--EIYQRHLMHESQTSYTLTNRQ---RINNVFEE---LMEASSRIKNL-PIKLFDY------------SS
Wildcat      MFSMELSKN--DLMSRIIAMAARVPYKELNSR--RLAAETRGK---VSQWISAAQNL-KFEVDDR------------AD
D29          YFSADSDAF--TQISRSVSILSGWSLERSTRA---VREQSIEE------SIANDLDEIPIRFNYK------------AS
Che12        YFSADSDAF--TQLTRSVSITTGWSLERSARA---VRNEDLAE------VAG-DLDPIPIRFNYK------------AS
Msm DnaB     IFSLEMSKS--EIVMRLLSAEAKIKLGDMRSG--RMTDDDWTR---LARRMSEISEA-PLYIDDS------------PN
BxZ2         YFSADSDAF--TQLSRSLSILTGWSMEKSARA---VREGDLG-------DAEDEFRDIPIRFNYN------------AS
BxB1         YLSADSDAF--TQSTRILSMELGMPLAESARA---VREGQLPPQVLTWNAAPGNPHGIPIRLNYS------------AQ
244          VFSVEMDDV--EVFSRLLAAGTWSPLSQIIGR--SMENSTWER---VEEYLDTHKDL-PLEIHDD------------AS
Cjw1         VFSVEMDDV--EVFSRLLAAGTWSPLSQIIGR--SMENSTWER---VEEYLDTHKDL-PLEIHDD------------AS
Gladiator    YFSADSDAF--TQLSRSVSILSGQSLEHATRS---VREQDIDE------GIAASLDEIPIRFNYK------------AS
Trixie       YFSADSDAF--TQLSRSVSILSGWSLERSTRA---VREQSIDD------AVAAELDALPIRFNYK------------AS
First        YFSADSDAF--TQLSRSVSILSGWSLERSTRA---VRDMTIPD------EIANELDALPIRFNYK------------AS
Redrock      YFSADSDAF--TQISRSVSILSGCSLEKSARD---VREQNIEE------AVAAELDEIPIRFNYK------------AS
EricB        YFSADSDAF--TQLSRSVSILSGQSLEQSTRA---VREQKIDP------DIASELDDIPIRFNYK------------AS
Turbido      YFSADSDAF--TQLSRSVSILSGWSLERSTRA---VRDMTIPD------DIGNELDALPIRFNYK------------AS
Msm*         IFSLEMSKS--EIVMRLLSAEARVKLADMRSG--RLGDQDWTK---IARRMAEISSA-PLYIDDS------------PN
Vch RecA     CAFIDAEHA--LDPV----YAKKLG-----VN-----------------IDELLVSQPDTGEQA-------------L
Hpy RecA     CAFIDAEHA--LDVY----YAKRLG-----VD-----------------TENLLVSQPSTGEEA-------------L
Sau RecA     AAFIDAEHA--LDPE----YAQALG-----VD-----------------IDNLYLSQPDHGEQG-------------L
Msm RecA     AAFIDAEHA--LDPE----YAKKLG-----VD-----------------TDSLLVSQPDTGEQA-------------L
Bam RecA     AAFIDAEHA--LDPV----YAQKLG-----VN-----------------IEELLLSQPDTGEQA-------------L
Eco RecA     CAFIDAEHA--LDPI----YARKLG-----VD-----------------IDNLLCSQPDTGEQA-------------L
Pvu RecA     CAFIDAEHA--LDPI----YAKKLG-----VD-----------------IDNLLCSQPDTGEQA-------------L
Hin RecA     CAFIDAEHA--LDPI----YAAKLG-----VD-----------------VKELFVSQPDNGEQA-------------L
Osa DCM1     VAYIDTEGT--FRPERIVPIAERFG-MDANAV-----------------LDNIIYARAYTYEHQ-------------Y
Hsa DCM1     IIFIDTENT--FRPDRLRDIADRFN-VDHDAV-----------------LDNVLYARAYTSEHQ-------------M
Ehi DCM1     VAYIDTEGT--FRPERIAQIAERFG-VDQTAV-----------------LDNILIARAYTHEQQ-------------F
Ath DCM1     VAYIDTEGT--FRPDRIVPIAERFG-MDPGAV-----------------LDNIIYARAYTYEHQ-------------Y
Zma DCM1     VAYIDTEGT--FRPERIVPIAERFG-MDANAV-----------------LDNIIYARAYTYEHQ-------------Y
Sce Rad51    CLYIDTEGT--FRPVRLVSIAQRFG-LDPDDA-----------------LNNVAYARAYNADHQ-------------L
Hsa Rad51    AMYIDTEGT--FRPERLLAVAERYG-LSGSDV-----------------LDNVAYARAFNTDHQ-------------T
Tbr Rad51    ALYIDTEGT--FRPERLVAVAERYS-LDPEAV-----------------LENVACARAYNTDHQ-------------Q
Bmo Rad51    CMYIDTEGT--FRPERLLAVAQRYG-MEGAAV-----------------LDNVAYARAYNTDHQ-------------T
Mmu Rad51    AMYIDTEGT--FRPERLLAVAERYG-LSGSDV-----------------LDNVAYARGFNTDHQ-------------T
Xla Rad51    AMYIDTEGT--FRPERLLAVAERYG-LSGSDV-----------------LDNVAYARAFNTDHQ-------------T
Eco Sms      TLYVTGEE----SLQQVAMRAHRLG------------------------LP--TDNLNMLSETS-------------I
Bsu Sms      VLYISGEE----SVKQTKLRADRLG------------------------IN--NPSLHVLSETD-------------M
Mtu Sms      ALYVSGEE----SAGQIRLRADRIG------------------------CGTEVEEIYLAAQSD-------------V
Ape RadA     AVYVDTEGT--FRWERIEQMARGVG-LDPDEV-----------------MKNIYWIRAINSHHQ-------------I
Hpy Sms      VLYVSGEE----SLSQIKMRAIRLD------------------------CI--EKELYLLNEIN-------------W
Tpa Sms      VLYVSGEE----SPGQIRGRADRLN------------------------IP--IQNIELLCATR-------------V
Eco DnaB     IFSLEMPSE--QIMMRSLASLSRVDQTKIRTG--QLDDEDWAR---ISGTMGILLEKRNIYIDDS------------SG
Bsu DnaB     IFSLEMGAE--QLVMRMLCAEGNINAQNLRTG--NLTEEDWGK---LTMAMGSLSNS-GIYIDDT------------PG
Mtu DnaB     IFSLEMSKS--EIVMRLLSAEAKIKLSDMRSG--RMSDDDWTR---LARRMSEISEA-PLFIDDS------------PN
Afu RadA     VIIIDTENT--FRPERIIQMAEAKG-LDGNEV-----------------LKNIYVAQAYNSNHQ-------------M
Mja RadA     AVYIDTEGT--FRPERIVQMAEALG-LDGNEV-----------------LNNIFVARAYNSDMQ-------------M
Mth RadA     AVFIDTENT--FRPERIEQIANAFE-LDLEEV-----------------LNKIHIARAFNSSHQ-------------I
Sis RadA     AVYIDTEGT--FRWERIENMAKALG-LDIDNV-----------------MNNIYYIRAINTDHQ-------------I
Mth RadB     TVFIDTEGG--LSVERIRQVSGDIF----DRV-----------------ADSIIVFEPSSFTEQ-------------G
Mja RadB     VIYIDTEGG--LSIERIKQIASNNY----KIV-----------------LENMIIYNAFDYEQ--------------D
Mma RadB     VVYIDTEGS--LSIERIKQLSEQDS----DEL-----------------LKSIIIYEPSSFEEQ-------------S
Afu RadB     VAYIDTEG---LSGERVRQIFGD------ERL-----------------FSNVFVYEVYRFRQQ-------------G
Hsa RadB     AVYVDTEG---LSLARFQQLLEARA-TDPEAA-----------------SANVIVSDAHDFDEQ-------------A
Pfu RadB     VAYVDTEGG--FSPERLAQMAESRN-LDVEKA-----------------LEKFVIFEPMDLNEQ-------------R
Hvo RadB     VVYIDTEG---LSVDRFQQLAEAVAPEDVESV-----------------TSRLMISEAYDFEDQ-------------E
Tga RadB     VAYVDTEGG--FSPERLSQMASARG-LDPEEA-----------------LSRFLLFTPGDIREQ-------------R
Mma KaiC     CLMIATEET----PEDILYQAELLG-HDIKPY-----------------YESGQLVIENIFESR-------------S
Sel KaiC1    GVFVTFEE----TPQDIIKNARSFG-WDLAKL-----------------VDEGKLFILDASPDP-------EGQEVVGG
```

```
Sel kaiC2   AILFAYEE----SRAQLLRNAYSWG-MDFEEM-----------------ERQNLLKIVCAYPES--------------
Mth KaiC1   GVMVSFDE----AMENLIENFRSSD-RLLERL-----------------IDEGLLFIEDASRG--------MDPDAGS
Mth KaiC2   CLFFSNEE----PADQIVRNMESIG-IKLGEF-----------------LGDKLL-IHSDRPTS--------------
Sso aRadC   CIYVTTEE----SRDSIIRQAKQFN-WDFEEY-----------------IEK-KLIIIDALMK-----EKEDQWSLVNL
Pae aRadC   GVFVALEE----HPVAVRRSFRHFG-WDIAQY-----------------EREGKFAIVDAFTGGVGTAAQRERYIVKQV
Pca aRadC   GLYITIDEPS-EDVKRGVRESLGWDLDALESQ------------NKLVFVDLRTHFRTYAKEEK------------VS
Tma aRadC   GIYVALEE----HPVQVKKNMEVFG-WNVDPF-----------------EKEGKFAIVDAFTGGIGEYAEKEKYVVRDI
Tko aRadC   GIYVALEE----HPVQVRQNMAQFG-WDVRKY-----------------EEEGLFAMVDAFTAGIGKSKEYEKYIVHDL
Ape aRadC   GVFVALEE----HPVQVRINMAQFG-WDVREY-----------------ERQGLFAVVDAFTSGIGEAAKKERYVVTDP
Hbu aRadC   NIYVTTEE----TRESIIKQAAMFG-FDFEKA-----------------IDDGKLVIIDALMG-----RHDDPWSLTEL
Sma aRadC   GIYVTTEE----SRESIIRQAAMFN-IDLRKY-----------------LDEGNLVIIDALME-----ERGDPWSLREL

                     170       180       190       200       210       220       230       240
            ....|....|....|....|....|....|....|....|....|....|....|....|....|....|....|....|
pRSF1010    IMAPEWFDGLKRAAEG-----RRLMVLDTLRRFHIEEENAS----------GPMAQVIGRMEAIAADT-GCSIVFLHHA
T4          GKMEKWREKST----------LGRLIVKQYPTGGADAN--------------TFRSLLNELKLKKNFVPTIIIVDYLGI
Gst DnaB    IRVSDIRAKCRRLKQESGL-G--MIVIDYLQLIQGSGRSKE-N-------RQQEVSEISRSLKALAREL-EVPVIALSQL
SPP1        QSVNYIWSKTRQTKRKNPG-KRVIVMIDYLQLLEP-AKAND-S-------RTNQISQISRDLKKMAREL-DVVVIALSQL
T7          AETDRLLAKLAYMRSGLG---CDVIILDHISIVVSASGESD---------ERKMIDNLMTKLKGFAKST-GVVLVVICHL
Mge DnaB    LTLQEIRNQITEVSKTSNV-R--LVIIDYLQLVNALKNNYGLT-------RQQEVTMISQSLKAFAKEF-NTPIIAAAQL
Wildcat     LTIEEIMQKCRIHKQRHGL-D--VVVIDYIQEVEESKGNSR---------EQ-AIAYIAKRARQIAKKL-DVVVIVAAQL
D29         PSLDEIENALAAYDALYED-FPALIVVDNITNVRTESGDGD-D-------PFSGLESLMDYLHEMARET-GSCVIGLHHV
Che12       PSLDVIEESLQAYDALYED-FPHLIVVDNITNVRTDSSDGD-D-------PFSGLESLMDYLHEMGRET-GACVVGLHHV
Msm DnaB    LTMMEIRAKARRLAQKSDL-R--LIVVDYMQLMTSGKKYES---------RQQEVSDFSRSLKLMAKEL-DVPVVAISQL
BxZ2        PSLDQIEASMMSYEEVYGD-FPALVVIDNVTNVRTGS-DED-D-------PFAGLESLMDYLHDMARST-SACVVGLHHV
BxB1        PTLKVIETSLAAYEETFGN-YPQLIVIDNITNVITGVAANDED-------PFGGLEVLMDWLHEKARET-GACIIGLHHV
244         LRVEEVVSRCRVRKPK-------VVMVDYAQLMENSNPKWD---------ERTNIAHISRSLKVAAKQL-HMVVVLASQL
Cjw1        LRVEEVVSRCRVRKPK-------VVMVDYAQLMENSNPKWD---------ERTNIAHISRSLKVAAKQL-HMVVVLASQL
Gladiator   PSLDEIENALTAYDALYED-FPALIVVDNITNVRTDSSDGD-D-------PFSGLESLMDYLHEMARET-GSCVIGLHHV
Trixie      PSLDVIEESLAAYDALYED-FPALIVVDNITNVRTESTEGD-D-------PFSGLESLMDYLHEMARET-GSCVIGLHHV
First       PSLDIIEESLEAYDALYED-YPALIVVDNITNVRTDAGEGD-D-------PFSGLESLMDYLHEMARET-GSCVIGLHHV
Redrock     PSLDIIEESLAAYDALYED-FPALIVVDNITNVRTDSSDGD-D-------PFSGLESLMDYLHEMGRET-GSCVIGLHHV
EricB       PSLDEIENALAAYDALYED-FPALIVVDNITNVRTDGGESD-D-------PFSGLESLMDYLHEMGRET-GSCVVGLHHV
Turbido     PSLDIIEESLEAYDALYED-YPALIVVDNITNVRTDNTDSD-D-------PFAGLESLMDYLHEMARET-GSCVIGLHHV
Msm*        LTMTEIRAKARRLKQKTDL-R--MVVVDYLQLMTSGKRVES---------RQAEVGEFSRSLKLLAKEL-DVPVIALSQL
Vch RecA    EICDALARSGA----------VDVIVVDSVAALTPKAEIEGEMGDSHMGLQARMLSQAMRKLTGNLKQS-NCMCIFINQI
Hpy RecA    EILETITRSGG----------IDLVVVDSVAALTPKAEIDGDMGDQHVGLQARLMSHALRKITGVLHKM-NTTLIFINQI
Sau RecA    EIAEAFVRSGA----------VDIVVVDSVAALTPKAEIEGEMGDTHVGLQARLMSQALRKLSGAISKS-NTTAIFINQI
Msm RecA    EIADMLVRSGA----------LDIIVIDSVAALVPRAEIEGEMGDSHVGLQARLMSQALRKMTGALNNS-GTTAIFINQL
Bam RecA    EIAEALVRSGA----------VDIVVVDSVAALVPKAEIEGDMGDSHVGLQARLMSQALRKLSGAINKS-KTIAIFINQI
Eco RecA    EICDALARSGA----------VDVIVVDSVAALTPKAEIEGEIGDSHMGLAARMMSQAMRKLAGNLKQS-NTLLIFINQI
Pvu RecA    EICDALTRSGA----------VDVIIVDSVAALTPKAEIEGEIGDSHMGLAARMMSQAMRKLAGNLKQA-NTLLIFINQI
Hin RecA    EICDALVRSGA----------IDVIIVDSVAALTPKAEIEGDMGDSHMGLQARLMSQALRKLTGQIKNA-NCLVVFINQI
Osa DCM1    NLLLGLAAKMA----EEP---FRLLIVDSVIALF-RVDFSGRG---ELAERQQKLAQMLSRLTKIAEEF-NVAVYITNQ-
Hsa DCM1    ELLDYVAAKFH----EEAG-IFKLLIIDSIMALF-RVDFSGRG---ELAERQQKLAQMLSRLQKISEEY-NVAVFVTNQM
Ehi DCM1    DLLIEVAARMA----EDH---FRMLIIDSVTSLF-RVDFSGRG---ELSERQQKLGKMMNKLIKI--------------
Ath DCM1    NLLLGLAAKMS----EEP---FRILIVDSIIALF-RVDFTGRG---ELADRQQKLAQML--------------------
Zma DCM1    NLLLGLAAKMA----EEP---FKLLIVDSVIALF-RVDFSGRG---ELAERQQKLAQMLSRLTKIAEEF-NVAVYITNQV
Sce Rad51   RLLDAAAQMMS----ESR---FSLIVVDSVMALY-RTDFSGRG---ELSARQMHLAKFMRALQRLADQF-GVAVVVTNQV
Hsa Rad51   QLLYQASAMMV----ESR---YALLIVDSATALY-RTDYSGRG---ELSARQMHLARFLRMLLRLADEF-GVAVVITNQV
Tbr Rad51   QLLLQASATMA----EHR---VAIIVVDSATALY-RTDYNGRG---ELAARQMHLGKFLRSLRNLANEY-NVAVVVTNQV
Bmo Rad51   QLLVQACAMMA----ESR---YSLIIVDSATALY-RTDYSGRG---ELNSRQLHLGRFMRMLLRLADEF-GVAVIITNQV
Mmu Rad51   QLLYQASAMMV----ESR---YALLIVDSATALY-RTDYSGRG---ELSARQMHLARFLRMLLRLADEF-GVAVVITNQV
Xla Rad51   QLLYQASAMMA----ESR---YALLIVDSATALY-RTDYSGRG---ELSARQMHLARFLRMLLRLADEF-GVAVVITNQV
Eco Sms     EQICLIAEEEQ----------PKLMVIDSIQVMHMADVQSSPG-------SVAQVRETAAYLTRFAKTR-GVAIVMVGHV
Bsu Sms     EYISSAIQEMN----------PSFVVVDSIQTVYQSDITSAPG-------SVSQVRECTAELMKIAKTK-GIPIFIVGHV
Mtu Sms     HTVLDQIETVQ----------PALVIVDSVQTMSTSEADGVTG-------GVTQVRAVTAALTAAAKAN-EVALILVGHV
Ape RadA    AIVDKLFTMVK-----ND--NIKLVVVDSVTSHF-RAEFPGRE---NLAMRQQLLNRHLHQLMRLADIF-NVAVVITNQV
Hpy Sms     PVIKANIESEN----------YFACVIDSIQTLYSPEISSAPG-------SISQVREITFELMRLAKTR-DIAIFIIGHI
Tpa Sms     EDVERVLNTRC----------PTFVIVDSIQTVFSPEAGAIPM-------TINQLKYCANELIGWVKER-DSVLFFTAHV
Eco DnaB    LTPTEVRSRARRIAREHGG-IG-LIMIDYLQLMRVPALSD-N--------RTLEIAEISRSLKALAKEL-NVPVVALSQL
Bsu DnaB    IRVSEIRAKCRRLKQESGL-G--MILIDYLQLIQGSGRSKD-N-------RQQEVSEISRELKSIAREL-QVPVIALSQL
Mtu DnaB    LTMMEIRAKARRLRQKANL-K--LIVVDYLQLMTSGKKYES---------RQVEVSEFSRHLKLLAKEL-EVPVVAISQL
Afu RadA    LLVDNAKELAEKL-KKEGR-PVRLIIVDSLMSHF-RAEYVGRG---TLADRQQKLNRHLHDLMKFGELY-NAAIVVTNQV
Mja RadA    LYAENVENLIR-----EGH-NIKLVIVDSLTSTF-RTEYIGRG---KLAERQQKLGRHMATLNKLADIY-NCVVIVTNQV
Mth RadA    LMAEKVNELIQ-----EGK-NIRLVIVDSLTAHF-RAEYVGRE---ALATRQQKLNQHLHTLQNIANTY-NAAVFVTNQV
Sis RadA    AIVDDLQELVS-----KDP-SIKLIVVDSVTSHF-RAEYPGRE---NLAVRQQKLNKHLHQLTRLAEVY-DIAVIITNQV
Mth RadB    EALQRTFSFLKTHG----D-STDLVLDSAVALY-RLKEGNAS------SFNLDLGRQMFLLLQMARRF-DLAAVITNQI
Mja RadB    KIIQKELPLITNN--------ASLIVVDNITSLY-RLELSDEAN--KNIMLNKMLGNQVKTLLKLAKTN-NLAVIITNQV
Mma RadB    EALEKIFFLEN----------IGLIVIDGIVSLY-RLELCDNIN--ENTKLNRMLGKQISNLLKVARMK-NSGILITNQV
Afu RadB    VAIQEAEKLCRSE-------KVKLVIVDCFTSLY-RSELEDDR---KQIKIKRELTSQLTFLLGMARKY-DVAVITNQM
```

```
Hsa RadB    QAVRDTADFADR---------ADLIVVDSVTGFY-RLARGGDD---TTGDALRQVADQITHLLSLARKH-DLAVVVTNQV
Pfu RadB    QVIARLKNIVNEK--------FSLVVVDSFTAHY-RAEGSRE---------YGELSKQLQVLQWIARRK-NVAVIVVNQV
Hvo RadB    QAVRDAEEFAAS---------ADLIVLDSATGFY-RLERTAEG---DGGEALRRVARQVTHLLSLARKH-DLAVVLTNQV
Tga RadB    RVIGSLKRLITPE--------FSLVVVDSITAHY-RAEEDWRS-------LTSELGKQLQVLLWIARKH-RIPVLVINQV
Mma KaiC    EIIGQTRYGFKPESLDIELPNLIEFIRDGTECLVIDNLGTFALR-----VPKKKLRDQFDGLNYLVRKKSCTTLLIMDES
Sel KaiC1   FDLSALIERINYAIQKY---RARRVSIDSVTSVFQQYDA-----------SSVVRRELFRLVARLKQI-GATTVMTTER
Sel kaiC2   AGLEDHLQIIKSEINDFK---PARIAIDSLSALARG-----VS--------NNAFRQFVIGVTGYAKQE-EITGLFTNTS
Mth KaiC1   YSLEALKLRLEDAIRRT---GARRVVLDKVDNLFDGTER-----------QGMIGFELRELIRWLNGM-GVTSVFTSG-
Mth KaiC2   LGLEAHLTYMQDLIMDFN---PDSVLVDPVTGLAGAGGSPETR--------NENAKHLFIRLTDFLKGR-GITSIFSYLI
Sso aRadC   T-PEELVNKVIEAKQKLGY-GKARLVIDSVSALFLDKP-------------AMARKISYYLKRVLNKW-NFTIYATSQY
Pae aRadC   DDVHELSDVLRQAIRDT---GARRVVIDSVSTLYLTKP-------------AVARGTIMTLKRVIAGL-GCTAFFVSQV
Pca aRadC   ADPRDIAKIILEYVKKFG---IKRLVIDPIAPLIITSHTD-----------VLWVREYMRELVFQLRKLKDVTTLMTSEI
Tma aRadC   DDVRELAEVLKRAVRET---QAKRVVIDSVTTLYITKP-------------AMARSIIFQLKRILSGL-GCTSLFVSQV
Tko aRadC   TDIREFIDVLRTAVKDI---GAKRLVVDSVTTLYINKP-------------AMARSIVMQLKRVLAGL-GVTSIFVSQI
Ape aRadC   EDVGLLIDVLKEAIRDV---GAKRVAVDSVSTLYLAKP-------------VLARRTVMLLKRVLSGL-GTTSILVSQV
Hbu aRadC   D-VETLVNKIIEAKRYLGY-GRARLVIDSMSAFWLDKP-------------AMARRYSYYVKRILARW-DFTALLTSQY
Sma aRadC   D-IEELLSKIIEAKKYLGY-GHARLVIDSMSAFWLDKP-------------AMARKYSYIVKRRLARW-NMTILLTSQY

                      250       260       270       280       290       300       310       320
            ....|....|....|....|....|....|....|....|....|....|....|....|....|....|....|....|
pRSF1010    SKGAAMMGAG-----DQQQASRGSSVLVDNIRWQSYLSSMTSAEAEEWGVDDDQRRFFVRFGVSKANYGAPFADRWFRRH
T4          CKSCRIR--------------VYSENSYTTVKAIAEELR--------ALAVETETVLWTAAQVGKQAW------------
Gst DnaB    SRSVEQRQDK----RPMMSDIRESGSIEQDADIVAFLYR--DDYYNKDSENKNI--IEIIIAKQRNGPVGTVQLA-----
SPP1        SRQVEQRQDK----RPMLSDLRESGQLEQDADIIEFLYR--DDYYDKESESKNI--VEVIIAKHRDGPVGTVSLA-----
T7          KNPDKGKAHEE-GRPVSITDLRGSGALRQLSDTIIALERNQQGDMPNLVLVRILKCRFTGDTGIAGYMEYNKETGWLEPS
Mge DnaB    SRRIEERKDS----RPILSDLRESGSIEQDADMVLFIHRTNDDKKEQEEENTNLFEVELIILEKNRNGPNGKVKLN-----
Wildcat     NRNLEDPKGK--PRLPVKSDFRESGGIENVADVAMILSR----TVDKDGNESKIPKMNLTFVKNRQGEERTIQLL-----
D29         TGPYNDGDKA----IPLGG---IKGQIGRVPEMVLTLHR----ESDGFGPDS----LNVSTVKNRGGKSDPSGND-----
Che12       TGPFNDGDKP----IPLSG---IKGQIGRVPEMVLTLHR----VSDGFGPDS----LNVSTVKNRGGKSDASGQD-----
Msm DnaB    NRGPEQRTDK----RPQVSDLRESGCMTANTRILRADTG--AEVTFGELMATGE--RPLVWSLDEHKRMVARPMT-----
BxZ2        TGGYNDADKA----IPLSG---VKGQISRVPEMILTLHR----VSEQFGSDS----LRVSTVKNRGGRSDPSGLD-----
BxB1        TADNNSGDKP----IPLSG---IKGQIGRVPELVATLHR----VPSTFGGDT----LRVSVVKNRSGRADPSGRL-----
244         NRENTKSTAKNGPAIPDLTNLAGGDSLGRDADVVLLLQK----ADDN------IIRMHVE--KNRNGRTGMIELV-----
Cjw1        NRENTKSTAKNGPAIPDLTNLAGGDSLGRDADVVLLLQK----ADDN------IIRMHVE--KNRNGRTGMIELV-----
Gladiator   TGQYNDGDKP----IPLGG---IKGQIGRVPEMVLTLHR----TSDGFGPDT----LNVSTVKNRGGKSDPSGQDFAALE
Trixie      TGPYNDGDKP----IPLSG---IKGQIGRVPEMVLTLNR----VSDGFGPDH----LNVSTVKNRGGKSDASGQDFASLE
First       TGPYNDGDKP----IPLSG---IKGQIGRVPEMVLTLNR----VSDGFGPDS----LNVSTVKNRGGKSDASGQDFASLE
Redrock     TGQYNDGDKP----IPLSG---IKGQIGRVPEMVLTLHR----VSDGFGPDS----LNVSTVKNRGGKSDPSGQDFASLE
EricB       TGQHNNGDSP----IPLGG---IKGQIGRVPEMVLTLHR----TSDGYGPDT----LNVSTVKNRGGKSDASGQDFASLE
Turbido     TGPYNDGNKP----IPLSG---IKGQIGRVPEMVLTLNR----VSDGFGPDS----LNVSTVKNRGGKSDASGQDFASLE
Msm*        NRGPEQRVDK----TPQLSDLRESGNLEQDCDLAILINR--PDANDRDHPRTGG--AYLHLAKNRHGQTRTCTVA-----
Vch RecA    RMKIGVM--------FGNPETTTGGNALKFYASVRLDIRRTGAIKEGEEVVGNETRIKVVKNKIAAPFKEANT-------
Hpy RecA    RMKIGMTG-------YGSPETTTGGNALKFYASVRIDIRRIAALKQNEQHIGNRAKAKVVKNKVAPPFREAEF-------
Sau RecA    REKVGVM--------FGNPETTPGGRALKFYSSVRLEVRRAEQLKQGQEIVGNRTKIKVVKNKVAPPFRVAEV-------
Msm RecA    REKIGVM--------FGSPETTTGGKALKFYASVRLDVRRIETLKDGTDAVGNRTRVKVVKNKVSPPFKQAEF-------
Bam RecA    REKVGVM--------FGNPETTPGGRALKFYSSVRLEVRRAEQLKQGNDVMGNKTRIKVVKNKVAPPFRTAEV-------
Eco RecA    RMKIGVM--------FGNPETTTGGNALKFYASVRLDIRRIGAVKEGENVVGSETRVKVVKNKIAAPFKQAEF-------
Pvu RecA    RMKIGVM--------FGNPETTTGGNALKFYASVRLDIRRTGAIKDGEEVVGSETRVKVVKNKVAAPFKQAEF-------
Hin RecA    RMKIGVM--------FGNPETTTGGNALKFYSSVRLDIRRTGSVKDGENIIGNETRVKVVKNKLAAPFRQVDF-------
Osa DCM1    --------------------------------------------------------------------------------
Hsa DCM1    TADPGATMTF----------------------------------------------------------------------
Ehi DCM1    --------------------------------------------------------------------------------
Ath DCM1    --------------------------------------------------------------------------------
Zma DCM1    IADPG---------------------------------------------------------------------------
Sce Rad51   VAQVDGGM-A----FNPDPKKPIGGNIMAHSSTTRLGFKKGKG-CQRLCKVVDSPCLPEA--------------------
Hsa Rad51   VAQVDGAA-M----FA----------------------------------------------------------------
Tbr Rad51   VANVDGAAPT----FQADSKKPIGGHIMAHASTTRLSLRKGRG-EQRIIKVYD---------------------------
Bmo Rad51   VAQVDSVG-V----FNA---------------------------------------------------------------
Mmu Rad51   VAQVDGAA-M----FA----------------------------------------------------------------
Xla Rad51   VAQVDGAA-M----FAADP-------------------------------------------------------------
Eco Sms     TKDGSLAG--------PKVLEHCIDCSVLLDGDADSRFRTLRSHKNRFGAVNELGVFAMTEQGL----------------
Bsu Sms     TKEGSIAG--------PRLLEHMVDTVLYFEGERHHTFRILRAVKNRFGSTNEMGIFEMREEGL----------------
Mtu Sms     TKDGAIAG--------PRSLEHLVDVVLHFEGDRNGALRMVRGVKNRFGAADEVGCFLLHDNGI----------------
Ape RadA    MARPDVFYGD----PTQ----AVGGHVLGHAPGVR---------------------------------------------
Hpy Sms     TKEGSIAG--------PRVLEHMVDSVLYFEGDPSRELRILRSFKNRFGPTSEIGLFEMKEQGLVSAKEASS--------
Tpa Sms     TKDGNIAG--------PKVVEHMVDTVISFERN-EEDIRFLRALKNRFGSVDELGIFTMGENGLSAVQDTAG--------
Eco DnaB    NRSLEQRADK----RPVNSDLRESGSIEQDADLIMFIYR--DEVYHENSDLKGI--AEIIIGKQRNGPIGTVRLT-----
Bsu DnaB    SRGVEQRQDK----RPMMSDIRESGSIEQDADIVAFLYR--DDYYDKETENKNI--IEIIIAKQRNGPVGTVSLA-----
Mtu DnaB    NRGPEQRTDK----KPMLADLRESGSLEQDADVVILLHR--PDAFDRDDPRGGE--ADFILAKHRNGPTKTVTVA-----
Afu RadA    MARPDVLFGD----PTK----PVGGHIVAHATATFRIYLKKGKD-DLRIARLIDSPHLPEGEAIFRVTERG---------
Mja RadA    AARPDALFGP----SEQ----AIGGHIVGHAATFRIFLRKAKG-DKRVAKLYDSPHLPDAEAMFRITEKG---------
```

```
Mth RadA    QARPDAFFGS----PTK----AIGGHVLGHAATYRIWLKKGLA-GKRIARLVDSPHLPEGECVFKITTAGIVD-------
Sis RadA    MARPDMFYGD----PTV----AVGGHTLYHVPGIRIQLKKSRG-NRRIARVVDAPHLPEGEVVFALTEEGIRDA------
Mth RadB    YSITGDDG--------REYVSPVGGTLLRYWSKVMVELEMGERPGERFAVLRRHRNRLEGSRVGFRIVADGIL-------
Mja RadB    RETVNG-------------FEASGGRLLEYWSKCIVRLEKLN--GDRLAILEKHLHAGE-ERVKFRIVERGIE-------
Mma RadB    KDSING-------------IEPAGGRLLEYWSKSIIKLEKTE--SIRKLTLEKHRHAKEGENLRFRIVQNGLE-------
Afu RadB    FTDVGSG-----------VDRPLGGPSLEHLSKVIIALERSN--ELRKATLIKHRWMKEGKSCFYRITDRGIE-------
Hsa RadB    FTDVDND---------SDRARPLGGHTLAHWTGTVLRLDRFRG-GTRRATLEKHRAKPDGEHAQFQITDGGID-------
Pfu RadB    YYDSNSG-----------ILKPIAEHTLGYKTKDILRFERLR-VGVRIAVLERHRFRPEGGMVYFKITDKGLE-------
Hvo RadB    YADPD-----------SDRTRALGGHTLEHWTGTVVRLDRFRG-GNRRATLEKHRAKPAGETATFKITDRGLS-------
Tga RadB    HFDARAE-----------RTRPVAEQTLGYRCKDILRLDKLPTPGKRVAILERHRFRPEGGMVYFKITEKGIE-------
Mma KaiC    AHNLTN--------QLAEYSVYGTIRLLVKENAYLGKMERYLCISKMRSTPISPDMSIFEITSEGIKIHE----------
Sel KaiC1   IEEYGP--IA----RYGVEEFVSDNVVILRNVLEGERRRRTLEILKLRGTSHMKGEYPFTITD-HGI-------------
Sel kaiC2   DQFMGAHSIT----DSHISTITDTIILLQYVEIRGE-MSRAINVFKMRGSWHDKAIREFMISD-KGP-------------
Mth KaiC1   --DYGG--SP----THRLEEYISDCVIHLTHTFEGQVGTRHLRIVKYRGSGHGLNRYPFIITR-RGA-------------
Mth KaiC2   ASPFTA-TTT----ELKLSSLIDTWIVLESIRANGE-YRRSLRILKSRGMNHSSSVAEVRFTD-RGILIKG---------
Sso aRadC   AITTSQ--AF----GFGVEHVADGIIRFRRMIRNGE-LHRYILIEKMRQTDHDKHVWEIDIVNGKGIVLK----------
Pae aRadC   SVGERG--FG----GPGVEHAVDGIIRLDLDEFDGR-LYRSIIVWKMRDTKHSMVRHPMEIKD-GGIEIMW---------
Pca aRadC   PTGENKIS------RFGVEEYLASGVIKLELMEYRGFVFRVMFIRKMRWTPVRPQKLVFEIYPHYGIYVLDRLENFMKQV
Tma aRadC   SVTEKG--FG----GPGVEHGVDGIIRLDLDEIDGE-LKRSLIVWKMRGTSHSMRRHPFEITD-KGIVIYP---------
Tko aRadC   SVGERG--FG----GPGVEHGVDGIIRLDLDEIDGE-LKRSLIVWKMRGTSHSMRRHPFDITD-KGIIVYP---------
Ape aRadC   SVTERG--FG----GPGVEHAADGIIRLDLDEVDGE-LVRSLIIWKMRGTKHSMRRHPFEITD-KGIIVYP---------
Hbu aRadC   AVTTSL--GF----GFGIEHVADGIIRFRKAVVRGE-LRRYVIIEKMRQTEHSLRMHEIEIRDGVGMRVK----------
Sma aRadC   AVTTSL--GF----GFGVEHVADGIIRFRKSVLGGE-LKRFLVVEKMRQTDHDKRVFLIDIVNEKGLVVL----------

                    330
            ....|....|...
pRSF1010    DGGVLKPAVL---
T4          -------------
Gst DnaB    -------------
SPP1        -------------
T7          -------------
Mge DnaB    -------------
Wildcat     -------------
D29         -------------
Che12       -------------
Msm DnaB    -------------
BxZ2        -------------
BxB1        -------------
244         -------------
Cjw1        -------------
Gladiator   FVGETMQINDFGL
Trixie      FVGDTMQINDFGH
First       FVGDTMQINDFGH
Redrock     FVGDTMQINDFR-
EricB       FVGETMQINDFGL
Turbido     FVGDTMQINDFGR
Msm*        -------------
Vch RecA    -------------
Hpy RecA    -------------
Sau RecA    -------------
Msm RecA    -------------
Bam RecA    -------------
Eco RecA    -------------
Pvu RecA    -------------
Hin RecA    -------------
Osa DCM1    -------------
Hsa DCM1    -------------
Ehi DCM1    -------------
Ath DCM1    -------------
Zma DCM1    -------------
Sce Rad51   -------------
Hsa Rad51   -------------
Tbr Rad51   -------------
Bmo Rad51   -------------
Mmu Rad51   -------------
Xla Rad51   -------------
Eco Sms     -------------
Bsu Sms     -------------
Mtu Sms     -------------
Ape RadA    -------------
Hpy Sms     -------------
```

```
Tpa Sms    -------------
Eco DnaB   -------------
Bsu DnaB   -------------
Mtu DnaB   -------------
Afu RadA   -------------
Mja RadA   -------------
Mth RadA   -------------
Sis RadA   -------------
Mth RadB   -------------
Mja RadB   -------------
Mma RadB   -------------
Afu RadB   -------------
Hsa RadB   -------------
Pfu RadB   -------------
Hvo RadB   -------------
Tga RadB   -------------
Mma KaiC   -------------
Sel KaiC1  -------------
Sel kaiC2  -------------
Mth KaiC1  -------------
Mth KaiC2  -------------
Sso aRadC  -------------
Pae aRadC  -------------
Pca aRadC  DMWYAS-------
Tma aRadC  -------------
Tko aRadC  -------------
Ape aRadC  -------------
Hbu aRadC  -------------
Sma aRadC  -------------
```

**S1 Figure.** Sequence alignment based on which the phylogenetic tree (Figure 1B) was constructed. Details of the abbreviations used are mentioned in the legend to Figure S2.
