# Supplementary material for: Biochemical Characterization of a Mycobacteriophage Derived DnaB Ortholog Reveals New Insight into the Evolutionary Origin of DnaB Helicases
Source: PLoS One. 2015 Aug 3;10(8):e0134762. doi: 10.1371/journal.pone.0134762 (PMC4523182; doi:10.1371/journal.pone.0134762)
Supplement: S2 Fig — (PDF) [file pone.0134762.s002.pdf]

S2 Figure

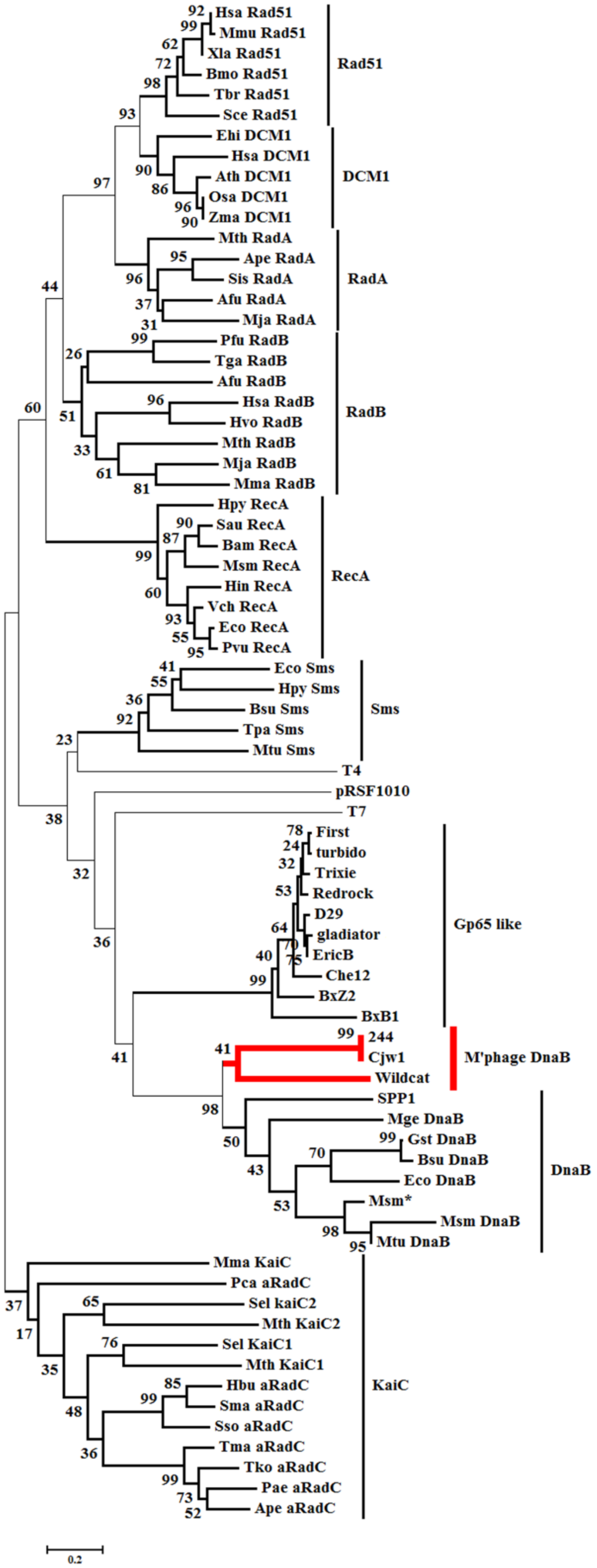

S2 Figure. Phylogenetic tree derived from an alignment of NTPase core domains of RecA superfamily members as indicated on the right (vertical bars). The Wildcat Gp80 cluster is highlighted in red. The evolutionary history was inferred using the Neighbor-Joining method.

The percentage of replicate trees in which the associated taxa clustered together in the bootstrap test (1000 replicates) is shown next to the branches. The tree is drawn to scale, with branch lengths in the same units as those of the evolutionary distances used to infer the phylogenetic tree. The arrow indicates the branch on which a root may be placed to convert the tree into a rooted one. Source organisms were identified by three-letter abbreviations except the phage ones. **(Afu)** *Archaeoglobus fulgidus;* **(Ape)** *Aeropyrum premix*; ;**(Ath)** *Arabidopsis thaliana;* **(Bam)** *Bacillus amyloticus*; **(Bsu)** *Bacillus subtilis;* **(Bmo)** *Bombix mori*; **(Eco)** *Escherichia coli*; **(Ehi)** *Entamoeba histolytica ;***(Gst)** *Geobacillus stearothermophilus*; **(Hsa)** *Halobacterium salinarum*; **(Hpy)** *Helicobacter pylori***;** **(Hbu)** *Hyperthermus butylicus*; **(Hsa)** *Homo sapiens*; **(Hin)** *Haemophilus influenzae***;** **(Mge)** *Mycoplasma genitalium*; **(Mja)** *Methanocaldococcus jannaschii*; **(Mth)** *Methanothermobacter thermoautotrophicus*; **(Mma)** *Methanosarcina mazei*; **(Mmu)** *Mus musculus* ; **(Mtu)** *Mycobacterium tuberculosis*, **(Msm)** *Mycobacterium smegmatis*; **(Msm*)** single domain RecA protein of *Mycobacterium smegmatis;* **(Osa)** *Oryza sativa* ; **(Pfu)** *Pyrococcus furiosus*; **(Pvu)** *Proteus vulgaris*; **(Pae)** *Pyrobaculum aerophilum*; **(Pca)** *Pyrobaculum calidifontis;* **(Sel)** *Synechococcus elongates*; **(Sis)** *Sulfolobus islandicus*; **(SPP1)** G40P of bacteriophage SPP1; **(Sau)** *Staphylococcus aureus;* **(Sma)** *Staphylothermus marinus*; **(Sce)** *Saccharomyces cerevisae*; **(Tbr)** *Trypanosoma brucei* ;**(Tko)** *Thermococcus kodakarensis*; **(Tga)** *Thermococcus gammatolerams*; **(Tma)** *Thermotoga maritima*; **(Tpa)** *Treponema pallidum*; **(Vch)** *Vibrio cholerae* ; **(Xla)** *Xenopus laevis*; **(Zma)** *Zea mays*; **(244)** Gp82 of Mycobacteriophage 244; **(CjW1)** Gp82 of Mycobacteriophage CjW1; **(Wildcat)** Gp80 of Mycobacteriophage Wildcat; **(BxB1)** Gp57 of Mycobacteriophage BxB1; **(BxZ2)** Gp64 of Mycobacteriophage BxZ2; **(D29)** Gp65 of Mycobacteriophage D29; **(Che12)** Gp71 of Mycobacteriophage Che12; **(T4)** Gp41 of *E.coli* phage T4; **(pRSF1010)** RepA of plasmid

pRSF1010, **(T7)** RecA core domain of *E.coli* phage T7 primase helicase; **(Gladiator)** Gp68 of Mycobacteriophage Gladiator; **(Trixie)** Gp67 of Mycobacteriophage Trixie; **(First)** DnaB like helicase of Mycobacteriophage First; **(Redrock)** Gp68 of Mycobacteriophage Redrock; **(EricB)** Gp69 of Mycobacteriophage EricB; **(Turbido**) Gp69 of Mycobacteriophage Turbido.
