# Supplementary material for: Biochemical Characterization of a Mycobacteriophage Derived DnaB Ortholog Reveals New Insight into the Evolutionary Origin of DnaB Helicases
Source: PLoS One. 2015 Aug 3;10(8):e0134762. doi: 10.1371/journal.pone.0134762 (PMC4523182; doi:10.1371/journal.pone.0134762)
Supplement: S3 Fig — (PDF) [file pone.0134762.s003.pdf]

S3 Figure

A

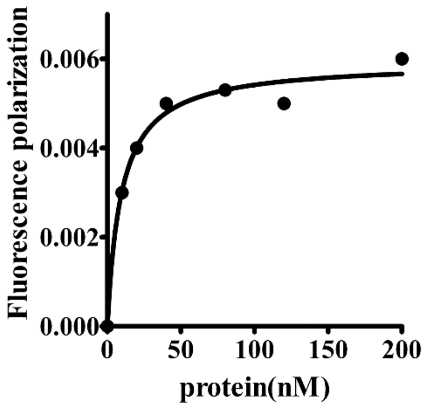

S3 Figure. Determination of binding constant (K_d) for the interaction between WCGp80 and the fork structure used in the helicase assays. The fork structure was generated by annealing oligonucleotides 1 and 5' 6 FAM labeled oligonucleotide 2 (S1 Table). Fluorescence polarization studies were performed in a Hitachi F-3010 spectro-fluorometer. The excitation and emission band passes were 5 nm. Fluorescence polarization measurements were done using a Hitachi polarizer accessory. The steady state fluorescence polarization (FP) was calculated according to the following equation:

$FP = I_{\parallel} - GI_{\perp} / I_{\parallel} + G I_{\perp}$……………………………eqn (i)

Where $I_{\parallel}$ is the intensity when the polarizers were in the same direction, $I_{\perp}$ is the intensity when the polarizers were crossed, and G is the grating factor that corrects for wavelength-dependent distortion of the polarizing system.

The titrations were performed by increasing the protein concentration in a stepwise manner. At each step, the FP was measured using the Eqn. i. The increase in FP upon addition of protein was plotted against protein concentration. The data points were curve fitted using an equation

representing a one site non-cooperative ligand binding model, $Y = \frac{Ymax.X}{Kd+X}$ Where Y is the increase in FP, X the protein concentration and K_d the dissociation constant and Ymax, the maximum increase in polarization.

Principle behind single turnover assay for helicase function:

An enzymatic reaction where an enzyme E catalyzes the conversion of a substrate S to its product P can be represented as follows.

$E + S = ES \rightarrow P$ -- eqn. ii

The dissociation constant K_d (which is the reciprocal of K_b, the binding constant) for the formation of the ES complex may be written as

$Kd = \frac{[E][S]}{[ES]}$. --- eqn. iii.

Let us consider that 120 nM of [E] is allowed to react with in 1nM of [S]. After the reaction attains equilibrium certain amount of ES will be formed the concentration of which may be assumed to be *x* nM. The equation can then be written as

$Kd = \frac{[120-x][1-x]}{[x]}$. --- eqn. iv

Since K_d is known (9.5 nM) from the fluorescence polarization experiment, it is possible to derive the value of *x* which turned out to be 0.95 nM. Hence almost all of the initially added substrate (1nM) is now bound to the enzyme resulting in the ES complex. The rate at which the ES complex decays is given by the first order rate equation

$[ES] = [ES°]e^{-kt}$ --- eqn. v

where [ES] and [ES°] represent the concentration of the ES complex at time t and zero respectively and k is the first order rate constant for the decay of the ES complex resulting in product formation. Product formed (unwound strands) will increase with the same rate constant and amplitude and will have an endpoint of $= [ES°]$. Thus if product formation is monitored then the equation will take the form

$[P] = [ES°](1 - e^{-kt})$ -------------------------------- eqn vi

where $[P]$ is the concentration at time t. Alternatively $[ES°]$ can be replaced by $[Pmax]$ since $[Pmax] = [ES°]$. The final form of the equation will be

$Y = [Ymax](1 - e^{-kt})$ ---------------------------------- eqn vii.

This equation was used to analyze the helicase activity under single turnover conditions resulting in the determination of the value of k, the first order rate constant for the breakdown of the ES complex into the products (Fig. 2F).
