# Supplementary material for: Biochemical Characterization of a Mycobacteriophage Derived DnaB Ortholog Reveals New Insight into the Evolutionary Origin of DnaB Helicases
Source: PLoS One. 2015 Aug 3;10(8):e0134762. doi: 10.1371/journal.pone.0134762 (PMC4523182; doi:10.1371/journal.pone.0134762)
Supplement: S4 Fig — (PDF) [file pone.0134762.s004.pdf]

**S4 Figure**

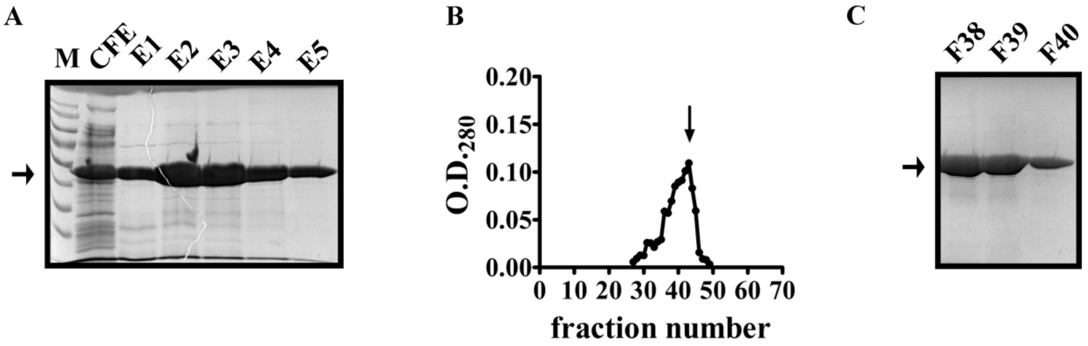


**S4 Figure**. **A.** The mutant protein WCGp80(K201A) also has a hexa-histidine tag like the wildtype protein WCGp80.It was purified by affinity chromatography in the same way as the wildtype. The figure shows 12% SDS-PAGE analysis of the affinity purified mutant protein WCGp80(K201A). The lanes marked CFE and E1-E5 represent cell-free extract and elution fractions respectively. **B.** The eluted fractions such as E2 and E3 were further purified using Size exclusion chromatography on a Sephacryl S200 gravity flow column. The mutant protein, like its wild-type counterpart, eluted in the void volume (arrow) **C.** The sample recovered after SEC was again analyzed on a 12% SDS-PAGE. In A and C arrows indicate the band corresponding to the purified protein which has a Mw of 52 kDa.
