# Supplementary material for: Biochemical Characterization of a Mycobacteriophage Derived DnaB Ortholog Reveals New Insight into the Evolutionary Origin of DnaB Helicases
Source: PLoS One. 2015 Aug 3;10(8):e0134762. doi: 10.1371/journal.pone.0134762 (PMC4523182; doi:10.1371/journal.pone.0134762)
Supplement: S5 Fig — (PDF) [file pone.0134762.s005.pdf]

S5 Figure

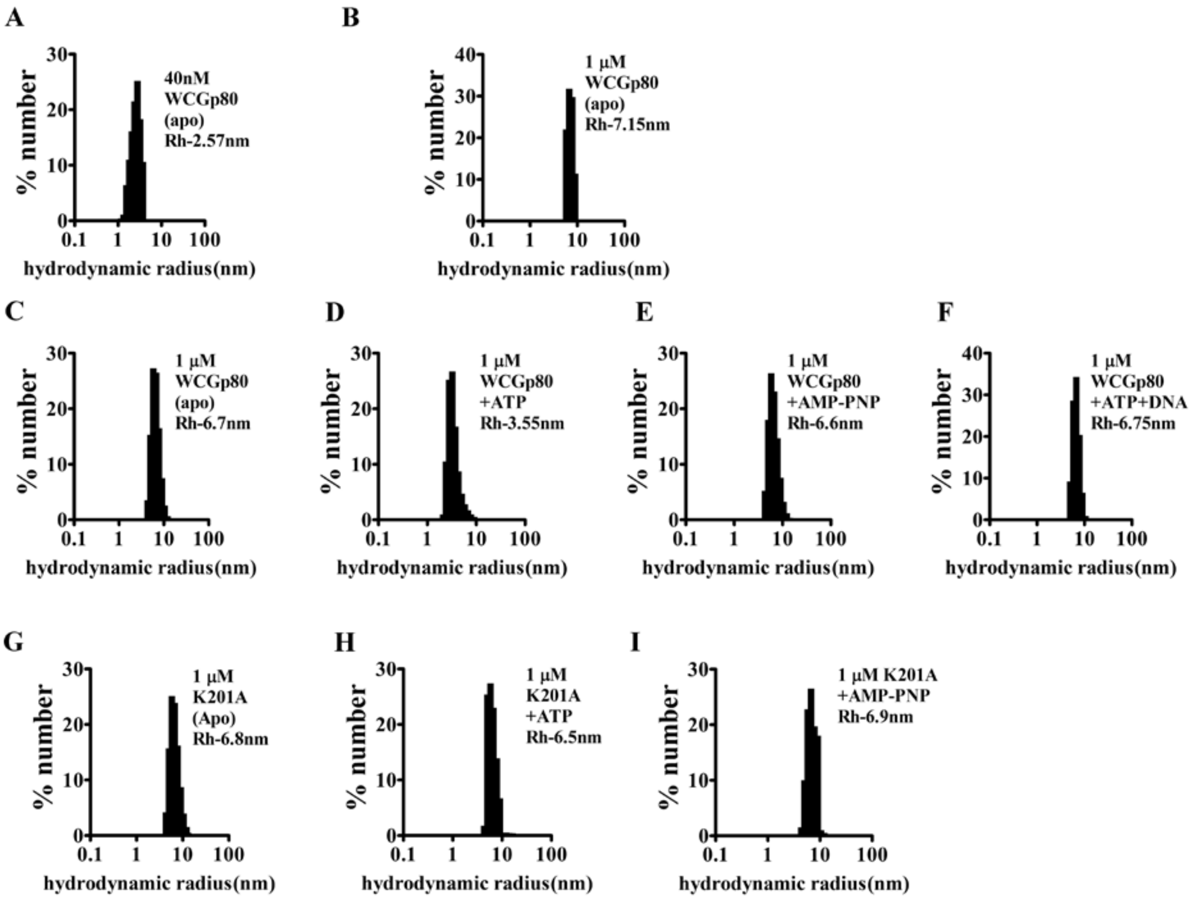

S5 Figure. . Distribution of hydrodynamic radii (R_h) of the native proteins either WCGp80 (A-F) or WCGp80(K201A) (G to I) as determined by performing DLS experiments. Experiments were performed in the absence of cofactors (apo enzyme) or in the presence of either ATP or AMP-PNP (4 mM) or DNA (100 nM) or combinations thereof. The concentrations of the proteins used were 1μM in all cases except for (A) where the protein was diluted to 40 nM. The panels shown represent the results of a single set of experiment. For quantitative interpretation, the weighted average of the R_h was determined. The results presented in Figs. 3, E and F are based on three such experiments.
