# Supplementary material for: Biochemical Characterization of a Mycobacteriophage Derived DnaB Ortholog Reveals New Insight into the Evolutionary Origin of DnaB Helicases
Source: PLoS One. 2015 Aug 3;10(8):e0134762. doi: 10.1371/journal.pone.0134762 (PMC4523182; doi:10.1371/journal.pone.0134762)
Supplement: S6 Fig — (PDF) [file pone.0134762.s006.pdf]

S6 Figure

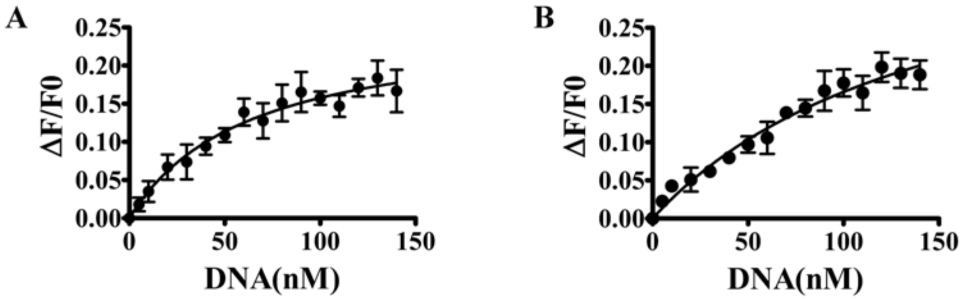

S6 Figure. Equilibrium binding of oligonucleotides to WCGp80. WCGp80 (40nM) was titrated with increasing amounts of 41-nt (A) and 60-nt (B) oligonucleotides. The samples were excited at 280 nm, and the fluorescence emission at 340 nm was measured. The excitation and emission slit widths were chosen to minimize bleaching and maximize the signal. The difference in the fluorescence values ΔF between the initial (F_0) and the final were noted and expressed as a fraction ΔF/F0, which represents the fractional change in fluorescence. The ΔF/F0 was plotted against DNA concentration. Curve fitting was done using a one site binding equation (eqn i) to obtain $K_{d..}$ The K_d values are 56.44 and 152 nM for 41 and 60-nt oligomers respectively.

$$Y = \frac{Bmax \times X}{Kd + X}$$ ------------------------------ (eqn i)
