# Supplementary material for: Biochemical Characterization of a Mycobacteriophage Derived DnaB Ortholog Reveals New Insight into the Evolutionary Origin of DnaB Helicases
Source: PLoS One. 2015 Aug 3;10(8):e0134762. doi: 10.1371/journal.pone.0134762 (PMC4523182; doi:10.1371/journal.pone.0134762)
Supplement: S7 Fig — Flat figure representation of Wildcat Gp80 alignment using ESPript 3.0.with PDB entry 3BH0. (PDF) [file pone.0134762.s007.pdf]

```
3BH0:A       ............................................................
EcDnaB       MAGNKPFNKQQAEPRERDPQVAGLKVPPHSIEAEQSVLGGLMLDNERWDDVAERVVADDF
HpyDnaB      ...................MDHLHKHLQQLQNIERIVLSGIVLANHKIEEIHSVLEPSDF
WildcatGp80  .........................MPAYDERAEQSVLGSILINPKCFGEIEG.LRSEHF
D29Gp65      ............................................................

3BH0:A

3BH0:A       ............................................................
EcDnaB       YTRPHRHIFTEMARLQESGSPIDLITLAESLERQGQLDSVGGFAYLAELSKNTPSAANIS
HpyDnaB      YYPPHGLFFEIALKLHEEDCPIDENFIRQKMPKDKQISEDDLVAIFAAS.....PIDNIE
WildcatGp80  FIPTHGYIFDLMQRMHAEDTELDAITIFAELDKEGKLAKVGGAGYLHDLLEAPKSPANIG
D29Gp65      ............................................................

3BH0:A                                                                  ooooo
                                 1         10            20             30
3BH0:A       ......................GSGPVKP.IQEAVSELMEIEASGTDDDDG..SIDEALV
EcDnaB       AYADIVRERAVVREMISVANEIAEAGFDPQGRTSEDLLDLAESRVFKIAESRANKDEGPK
HpyDnaB      AYVEEIKNASIKRKLFTLANTIREQALES.AQKSSDILNTVEREVYALLNG..SMIEGFR
WildcatGp80  YYAKVVMEKWKLR....TVNDIGTRLASLQTDDIEDIPILLERAHTFLDEIDAEDDSESA
D29Gp65      ............................................................

             α1                                  α2             β1
3BH0:A       ooooooo                             ooooooo      TT  --->  TT  ooo
                 40                   50            60           70          80
3BH0:A       TVYEEIESAD.........G..NITGVPSGFTELDRMTYG.YKRRNFVLIAARPSMGKTA
EcDnaB       NIADVLDATVARIEQLFQQPHDGVTGVNTGYDDLNKKTAG.LQPSDLIIVAARPSMGKTT
HpyDnaB      GIKEVLESAMNLITENQRKGSLKVTGIPTGFVQLDNYTSG.FNQGSLVIIGARPSMGKTS
WildcatGp80  DLRELYDGWLEWQAD.......DRPFIETPFLAVNDLLNGGMVRQRMLVVGARPGCGKTV
D29Gp65      ..............................................MVLVCAGPGTGKSA

                α3            β2            α4            α5     α6
3BH0:A       oooooooooo     ----->      ooooooooooooo     ooooo  oooo     oooo
                     90          100         110         120         130         140
3BH0:A       FALKQAKNMSDN.DDVVNLHSLEMGKKENIKRLIVTAGSINAQKIKAARRDFASEDWGKL
EcDnaB       FAMNLVENAAMLQDKPVLIFSLEMPSEQIMMRSLASLSRVDQTKIRTGQLD..DEDWARI
HpyDnaB      LMMNMVLSALND.DRGVAVFSLEMSAEQLALRALSDLTSINMHDLESGRLD..DDQWENL
WildcatGp80  FVSQLGFYAALQ.HLSVLMFSMELSKNDLMSRIIAMAARVPYKELNSRRLA..AETRGKV
D29Gp65      FVLAYALKSKVP....TLYFSADSDAFTQISRSVSILSGWSLERSTRAVRE......QSI

              α7          β3            α8                β4     η1
3BH0:A       oooooooo    --->      ooooooooooooo       ------>  ooo      TT
                   150         160         170         180         190         200
3BH0:A       SMAIGEISNS.NINIFDKAGQSVNYIWSKTRQTKRKNPGKRVIVMIDYLQLLEPAKANDS
EcDnaB       SGTMGILLEKRNIYIDDSSGLTPTEVRSRARRIAREHG.GIGLIMIDYLQLMRVPALSDN
HpyDnaB      AKCFDHLSQK.KLFFYDKSYVRMDQIRLQLRKLKSQHK.ELGIAFIDYLQLMSGNKATRE
WildcatGp80  SQWISAAQNL.KFEVDDRADLTIEEIMQKCRIHKQRHG..LDVVVIDYIQEVEESKGN.S
D29Gp65      EESIANDLDEIPIRFNYKASPSLDEIENALAAYDALYEDFPALIVVDNITNVRTESGDGD

                      α9                  β5        η2           η3     α10
3BH0:A       oooo.ooooooooooooooo     ------>    ooo          oooo   ooooo
                     210         220         230         240         250
3BH0:A       RTNQ.ISQISRDLKKMARELDVVVIALSQLSRQVEQRQDKR..PMLSDLRESGQLEQDAD
EcDnaB       RTLE.IAEISRSLKALAKELNVPVVALSQLNRSLEQRADKR..PVNSDLRESGSIEQDAD
HpyDnaB      RHEQ.IAEISRELKTLARELEIPIIALVQLNRSLENRDDKR..PILSDIKDSGGIEQDAD
WildcatGp80  REQA.IAYIAKRARQIAKKLDVVVIVAAQLNRNLEDPKGKPRLPVKSDFRESGGIENVAD
D29Gp65      DPFSGLESLMDYLHEMARETGSCVIGLHHVTGPYNDGDKAI..PLGG...IKGQIGRVPE

               β6               α11                                  β7
3BH0:A       ------->          oooo                       -.........------
             260         270                     280
3BH0:A       IIEFLYR.........DDYYDKE................SESKNI.........VEVIIA
EcDnaB       LIMFIYR.........DEVYHEN................SDLKGI.........AEIIIG
HpyDnaB      IVLFLYRGYIYQMRAEDNKIDKLKKEGKIEEAQELHLKVNEERRIHKQNGSIEEAEIIVA
WildcatGp80  VAMILSR...........TVDKDG...............NESKIP........KMNLTFV
D29Gp65      MVLTLHR.............ESD................GFGPDS.........LNVSTV

                         β8       η4   β9
3BH0:A       -->TT    -------->  ooo  --->
                 290         300         310
3BH0:A       KHRDGPVGTVSLAFIKEYGNFVNLERRFDDR............
EcDnaB       KQRNGPIGTVRLTFNGQWSRFDNYAGPQYDDE...........
HpyDnaB      KNRNGATGTVYTRFNAPFTRYEDMPMDSHLEEGQETKFEIPTT
WildcatGp80  KNRQGEERTIQLLERFDQQRFDNIFGSTTDDMRHNDEAVA...
D29Gp65      KNRGGKSDPSGNDYAALEFIGDTMQINDFDH............
```

S7 Figure. Structure based alignment of Wildcat Gp80 protein. Flat figure representation of Wildcat Gp80 alignment using ESPript 3.0.with PDB entry 3BH0: A (*Bacillus* phage G40P ATPase core). *E.coli* DnaB helicase, *Helicobacter pylori* DnaB and D29 Gp65 were also included. Secondary structure elements are presented on top.
