# Supplementary material for: Biochemical Characterization of a Mycobacteriophage Derived DnaB Ortholog Reveals New Insight into the Evolutionary Origin of DnaB Helicases
Source: PLoS One. 2015 Aug 3;10(8):e0134762. doi: 10.1371/journal.pone.0134762 (PMC4523182; doi:10.1371/journal.pone.0134762)
Supplement: S8 Fig — (PDF) [file pone.0134762.s008.pdf]

S8 Figure

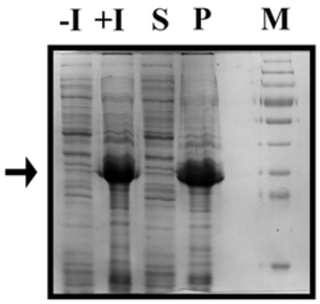

S8 Figure. 12% SDS-PAGE analysis of total protein extracted from *E. coli* cells synthesizing ΔN(1-189)WCGp80 from a recombinant vector based on the expression plasmid pET28a. Unlike the Thio-ΔN(1-189)WCGp80 recombinant, ΔN(1-189)WCGp80 does not possess a thioredoxin tag and is insoluble, (lane S) compared to (lane P). Lanes marked (I) either – or + refer to IPTG induced (+) or un induced (-) conditions. Lane M represents Mw markers. The arrow points to the band corresponding to the desired protein.
