# Supplementary material for: Biochemical Characterization of a Mycobacteriophage Derived DnaB Ortholog Reveals New Insight into the Evolutionary Origin of DnaB Helicases
Source: PLoS One. 2015 Aug 3;10(8):e0134762. doi: 10.1371/journal.pone.0134762 (PMC4523182; doi:10.1371/journal.pone.0134762)
Supplement: S9 Fig — The WCGp80 and Thio- ΔN(1–189)WCGp80 proteins were run in the same gel as controls. (PDF) [file pone.0134762.s009.pdf]

S9 Figure

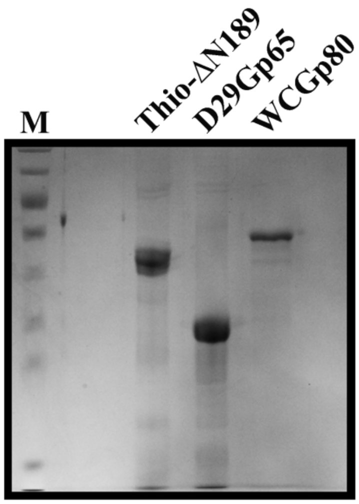

S9 Figure. 12% SDS-PAGE profile of Thio-ΔN189, D29Gp65 and WCGp80. Thio-ΔN189 is the abbreviation for Thio-ΔN(1-189)WCGp80.
