# Supplementary material for: Biochemical Characterization of a Mycobacteriophage Derived DnaB Ortholog Reveals New Insight into the Evolutionary Origin of DnaB Helicases
Source: PLoS One. 2015 Aug 3;10(8):e0134762. doi: 10.1371/journal.pone.0134762 (PMC4523182; doi:10.1371/journal.pone.0134762)
Supplement: S1 Table — (PDF) [file pone.0134762.s010.pdf]

	Substrate	Sequence
Unwinding	Forked 20 bp duplex with 40 ntd 5´ and 3´ tails	5’- GCC CTGATCACGGTACTCGGTTTTTTTTTTTTTTTTTTTTGGCTCCTCTAGACTCGACCG- 3’ (oligo1)
		5’- CGGTCGAGTCTAGAGGAGCCTTTTTTTTTTTTTTTTTTTTTTTTTTTTTTTTTTTTTTTT- 3’ (oligo2)
	ds 22 bp with 23 nt 5´ tail	5´-GTACCCGTGGATCCTCTAGAGT-3´
		5´-GTTATTGCATGAAAGCCCGGCTGACTCTAGAGGATCCACGGGTAC-3´
	ds 22 bp with 23 nt 3´ tail	5´-GTACCCGTGGATCCTCTAGAGT-3´
		5´-ACTCTAGAGGATCCACGGGTACGTTATTGCATGAAAGCCCGGCTG-3´
ATPase assay	7-mer	5´-GGCTCCTCTA-3´
	17-mer	5'-GTAAAACGACGGCCAGT-3'
	30-mer	5´-CCCGGTGTGGGTAAGACACTCGGGCTGGAC-3´
	41-mer	5'-TCGAGCTCGGTACCCGGGGATCCTCTAGAGTCGACCTGCAGG-3'
	60-mer	5’ CGGTCGAGTCTAGAGGAGCCTTTTTTTTTTTTTTTTTTTTTTTTTTTTTTTTTTTTTTT-3’
	100-mer	5´CGTGATCCAGGGTTCGGGAGACAACCGATGAAGCACGGAGGACCGCGCTGATGACCAATCAGTATCCG CCCCAACAAGATCGACCTCAGTGGTACGCGGG-3´

S1_Table

oligo 1: 3´ end of this oligomer was labeled with black hole quencher 1 for fluorescence based helicase assay.

oligo 2: 5´ end of this oligomer was labeled with γ^{32}P ATP for radioactive helicase assay or with 6-FAM for fluorescence based helicase assay.
